# Theoretical Studies on Mechanism of Inactivation of Kanamycin A by 4′-O-Nucleotidyltransferase

**DOI:** 10.3389/fchem.2018.00660

**Published:** 2019-01-29

**Authors:** Sergio Martí, Agatha Bastida, Katarzyna Świderek

**Affiliations:** ^1^Departament de Química Física i Analítica, Universitat Jaume I, Castelló de La Plana, Spain; ^2^Departamento de Química Bio-orgánica, Instituto de Química Orgánica General (CSIC), Madrid, Spain

**Keywords:** kanamycin, antibiotic, QM/MM, aminoglycosides, kinetic isotope effects, O-Nucleotidyltransferase, electrostatic effects, compression effects

## Abstract

This work is focused on mechanistic studies of the transfer of an adenylyl group (Adenoside-5′-monophosfate) from adenosine 5′-triphosphate (ATP) to a OH-4′ hydroxyl group of an antibiotic. Using hybrid quantum mechanics/molecular mechanics (QM/MM) techniques, we study the substrate and base-assisted mechanisms of the inactivation process of kanamycin A (KAN) catalyzed by 4′-O-Nucleotidyltransferase [ANT(4′)], an active enzyme against almost all aminoglycoside antibiotics. Free energy surfaces, obtained with Free Energy Perturbation methods at the M06-2X/MM level of theory, show that the most favorable reaction path presents a barrier of 12.2 kcal·mol^−1^ that corresponds to the concerted activation of O4′ from KAN by Glu145. In addition, the primary and secondary ^18^O kinetic isotope effects (KIEs) have been computed for bridge O3α, and non-bridge O1α, O2α, and O5′ atoms of ATP. The observed normal 1°-KIE of 1.2% and 2°-KIE of 0.07% for the Glu145-assisted mechanism are in very good agreement with experimentally measured data. Additionally, based on the obtained results, the role of electrostatic and compression effects in enzymatic catalysis is discussed.

## Introduction

Aminoglycoside antibiotics (AGAs) belong to the class of agents used to treat serious infections caused by bacteria that either multiply very quickly or are difficult to treat. Their role is to stop bacteria from producing proteins needed for their survival (Shaw et al., 1993). Unfortunately, due to widespread usage of these antibiotics in clinical treatment, bacterial strains have appeared that make these compounds ineffective. In fact antibiotic-resistant bacterial infection has become a concerning global threat to human health, according to World Health Organization (WHO) reports (Nature, 2013; Berendonk et al., 2015).

Several mechanisms of resistance to AGAs have been proposed including (a) the presence of AGA-modifying enzymes (Ramirez and Tolmasky, 2010), (b) the decrease of bacteria membrane permeability toward AGA uptake into the bacteria and extrusion of AGAs from the cell by efflux pumps (Kumar and Schweizer, 2005), and (c) the modification of the drug target as a result of mutations in the ribosome (Pfister et al., 2003) or methylations by 16S rRNA methyltransferases influencing the binding of AGAs (Doi and Arakawa, 2007; Leban et al., 2017). Nevertheless, the major mechanism of bacterial resistance in clinical isolates of gram-negative and gram-positive bacteria is assigned to enzymatic modification of the amino or hydroxyl groups of AGAs (Vakulenko and Mobashery, 2003).

**Figure 1:**
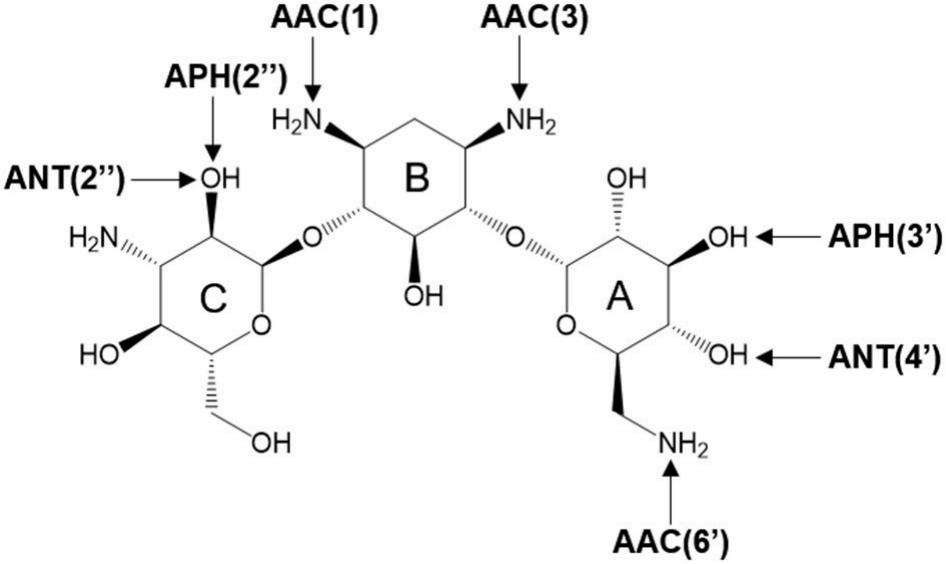
Sites of possible modification on kanamycin A by various AGAs-modifying enzymes. The arrows point to the sites of modification by acetyltransferases (AACs), phosphotransferases (APHs), and nucleotidyltransferases (ANTs).

**Figure 2:**
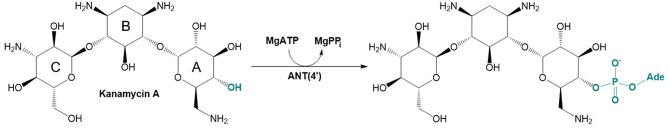
Reaction catalyzed by aminoglycoside O-adenylyltransferase ANT(4′) in MgATP-dependent process of 4′-O-adenylation of kanamycin A.

There are three different families of AGA-modifying enzymes: the acetyl-CoA-dependent aminoglycoside acetyltransferases (AACs), the ATP-dependent aminoglycoside phosphotransferases (APHs), and the ATP-dependent aminoglycoside nucleotidyltransferases (ANTs) (Becker and Matthew, 2013). Sites of modification in kanamycin A (KAN) by different AGA-modifying enzymes are indicated on Figure 1. Each of these families catalyzes a different type of reaction. Thus, APHs catalyze regiospecific transfer of γ-phosphoryl group of ATP to one of the hydroxyl substituents presented in AGAs, AACs facilitate N/O-acetylation groups in AGAs, and finally ANTs promote reaction between MgATP and AGAs allowing to form O-adenylylated aminoglycoside and the magnesium chelate of inorganic pyrophosphate (MgPP_i_) formed after the reaction, as presented in Figure 2.

Herein, we focus on mechanistic studies of reaction catalyzed by 4′-O-Nucleotidyltransferase from *S. aureus*, which modifies KAN antibiotic in the 4′-OH [ANT(4′)] position. KAN is a second-line injectable drug used in the treatment of tuberculosis (TB) (Ventola, 2015). TB is classified among 18 of the most serious drug-resistant threats^1^, since in many cases, as reported by WHO, TB can be resistant to first-line drugs (600,000 cases in year 2016) and/or to any other treatment (490,000 with multidrug-resistant TB). The role of KAN is to interact with the 30S subunit of prokaryotic ribosomes (exactly to bind in 16S rRNA, at the tRNA acceptor A site) in order to substantially rise the amounts of mistranslation (incorrect alignment with the mRNA), and it indirectly inhibits translocation that provokes insertion of the wrong amino acid into the peptide during protein synthesis (Pestka, 1974; Misumi and Tanaka, 1980; Carter et al., 2000). KAN belongs to an AGA family containing a 4,6-disubstituted 2-deoxystreptamine core (B ring) glycosidically linked at the 4 position to a glucosaminopyranose (ring A) and at position 6 to a N-acetylglucopyranose (ring C), as shown on Figure 1.

**Figure 3:**
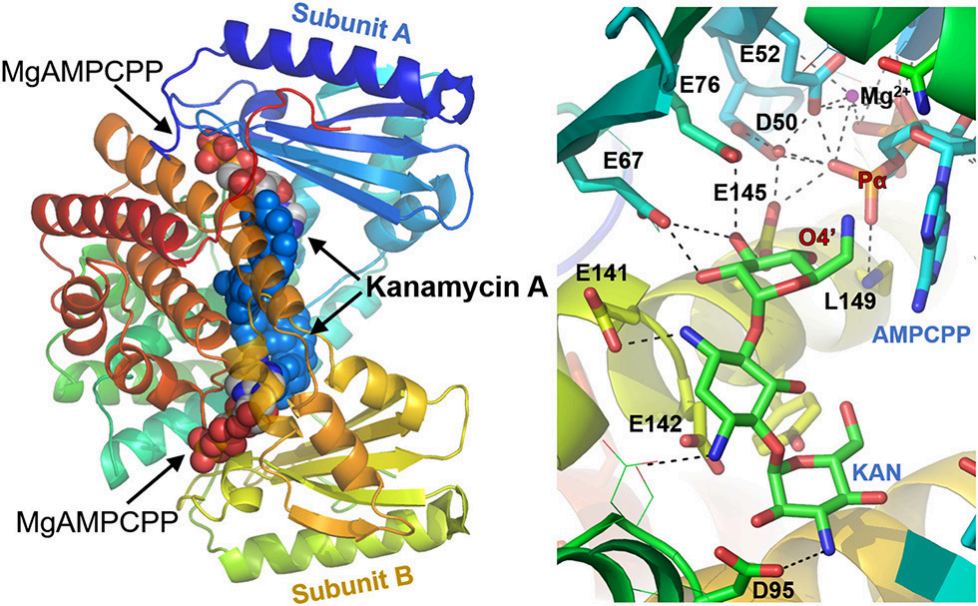
Structure of ANT(4′) homodimer from *Staphylococcus aureus* (pdb:1kny) with bound AMPCPP complexed with Mg^2+^ cation and Kanamycin A in both active sites **(Left)**, together with detailed representation of the active site **(Right)**.

The crystal structures of the D80Y variant of ANT(4′) with bound KAN and AMPCPP (adenosince-5′-[(α,β)-methylene]triophosphate) indicate that the active form of the enzyme is a homodimer (Pedersen et al., 1995). Each monomer binds both substrates, nucleotide and aminoglycoside (KAN). However, and interestingly, the reaction takes place between substrates that are bound to different subunits. In general, as shown in Figure 3, the two nucleotides are far apart, and the two KAN molecules are at a distance of 3.5 Å from each other. The amino acid residues from the N-terminal domain interact mostly with the triphosphate moiety of the nucleotide, while those from C-terminal with the KAN (Revuelta et al., 2010).

Based on the analysis of the organization of the active site, it is believed that in order to access their binding pockets, both substrates must diffuse via the same cavity characterized by a strong negative electrostatic potential (Matesanz et al., 2012). Thus, it is assumed that KAN should bind before the nucleotide, reducing in this way possible repulsive interaction by an anionic specie (ATP) and highly charged regions of protein (Chen-Goodspeed et al., 1999). This is in contradiction to the studies previously done for ANT(2′), where an inverse order of binding was suggested (Lombardini and Cheng-Chu, 1980; Gates and Northrop, 1988). Nevertheless, the presence of both substrates is required for catalysis to take place.

The results of previous kinetic studies (Pelt et al., 1986) of *S. aureus* ANT(4′), with gentamicin as a substrate, indicate that the mechanism of antibiotic inactivation involves direct nucleotidyl transfer from ATP to the aminoglycoside. In fact, what was exactly observed experimentally, is inversion of stereochemistry on the α-phosphorus atom, suggesting that the chemical step involves an uneven number of phosphotransfers. Additionally, a concerted and slightly associative structure of transition state (TS) for the enzymatic 4′-adenylation of KAN was suggested from the measurements of ^18^O kinetic isotope effects (KIEs) for key oxygen atoms (Gerratana et al., 2001).

The transferred group in this reaction is the nucleoside monophosphate (see Figure 2), which is a monosubstituted derivative of the phosphoryl group, and it can be assumed that its chemical nature along the reaction path is similar to the non-substituted relative. Thus, as it is well known, phosphoryl transfer reactions with low-lying d-orbitals on the phosphorus atoms that permit the existence of phosphorus pentavalent moiety as intermediate (Cleland and Hengge, 2006; Marcos et al., 2008; Kamerlin, 2011; Wymore et al., 2014) have been proposed to proceed by two alternative mechanisms (Kamerlin et al., 2008), i.e., associative, where nucleophilic attack takes place before the living group departures, and dissociative, in which withdrawal of the living group preludes the nucleophilic attack. The reaction can proceed via only one step in which asynchronous forming and breaking bonds can still be observed since associative-like or dissociative-like mechanisms can be found for concerted paths (Bordes et al., 2017).

The degree to which nucleotidyl transfer goes by an associative mechanism is difficult to determine, since experimental characterization of TS geometry is not feasible. The nature of the TS for the non-enzymatic (Zhang et al., 2014) and enzymatic (Sucato et al., 2007, 2008; Oertell et al., 2012, 2014) nucleotidyl transfer reaction has recently been studied in human DNA polymerase β using different modified dNTP substrates that differed in the chemical structure.

In this paper, insight into the nucleotidyl transfer mechanism for KAN inactivation by ANT(4′) at molecular level is done using a QM/MM approach (Ridder and Mulholland, 2003). Molecular details of reactions involving ATP-cofactor are essential for understanding the mechanism phosphoryl transfer reactions promoted by presence of metals in the living cell, responsible for a wide range of processes (Cherepanov et al., 2008). In order to obtain values which can be directly compared with experimental data, the free energy surfaces were computed and compared to experimentally measured rate constants using the transition state theory (TST). Moreover, the ^18^O kinetic isotope effects (KIEs) for bridge and non-bridge oxygen of ATP were determined and compared with experimental values. Finally, electrostatic potential generated by ANT(4′) in the active site was computed and compared with non-catalyzed reaction in order to understand the role of electrostatic effects for this particular reaction.

## Computational Methods

### System Setup

A molecular model of aminoglycoside 4'-nucleotidyltransferase [ANT(4′)] was built based on biological assembly of homodimer structure with PDB ID 1KNY (Pedersen et al., 1995). The X-ray structure contains two KAN molecules bound into both active sites present in the enzyme, together with the non-hydrolizable ATP-cofactor, AMPCPP-Mg^2+^ ion. In order to modify AMPCPP to ATP, the carbon atom was changed by oxygen in the α3-position. Thus, the final enzymatic model consisted of two subunits (253 residues each), two Mg^2+^ chelate of ATP, and two KAN molecules.

Missing hydrogen atoms were added to the enzyme structure using the tLEAP (Schafmeister et al., 1995) program based on results of pKa computed for titratable residues at pH 7 using the PropKa 3.1 program (Olsson et al., 2011; Sondergaard et al., 2011). Results of pKa calculations are presented in Table S1. Interestingly, all glutamic acid residues for which the predicted values of pKa were over 7 are involved in the strong H-bond interactions with KAN (Glu67 ring A; Glu76 ring A; Glu142 ring C) and they should not be protonated. This misleading result is an effect of absence of hydrogen atoms in the substrate once the pKa values are computed. For the rest of the titratable residues, standard values of pKa were obtained. The geometrical analysis of specific interactions involving histidine residues were done and the following protonation states for these residues were assigned: for His17, 66, 181, and 241 the nitrogen atom of imidazole ring in δ-position was protonated, while in case of His100, 180, 181, and 207, hydrogen was added to the nitrogen atom in ε-position. Finally, no S-S bridge between Cys residues was detected. The kanamycin molecule was assumed to be a neutral species with the total charge set to 0. All hydroxyl groups were protonated, and all amine groups were assigned to be neutral.

Subsequently, neutralization of the system was achieved by adding 40 Na^+^ counterions in the electrostatically most favorable positions. Finally, a full enzyme model was solved in a 10 × 10 × 10 nm^3^ cubic box of TIP3P (Jorgensen et al., 1983) water. Then, 10 ns of NVT molecular dynamic (MD) simulations with a time step of 1 fs at 300 K were carried out after prior optimization, heating (from 0 to 300 K with 0.001 K temperature increment) and equilibration processes of 100 ps using an AMBER (Duan et al., 2003) force field implemented in NAMD (Phillips et al., 2005) software. The temperature during the MD simulation was controlled using the Langevin thermostat (Grest and Kremer, 1986). In order to improve the time of simulations, cut-offs for non-bonding interactions were applied using a smooth switching function between 14.5 and 16 Å. Additionally, periodic boundary conditions (PBC) were used. The parameters developed previously by Carlson and co-workers were used to describe the ATP-cofactor at MM level (Meagher et al., 2003). Missing parameters for KAN were generated using the antechamber tool and GAFF (Wang et al., 2004), with charges computed at Austin Model 1 (AM1) (Dewar et al., 1985) level. The obtained parameters are presented in Table S2. Root-mean-square-deviation (RMSD) and more analysis of MD simulations are provided in Supplementary Material. According to the time-dependent evolution of the RMSD for the position of C-Cα-N, atoms of the protein backbone, the system can be considered equilibrated (see Figure S1).

### Potential Energy Surfaces

The last structure of MD simulation was then employed for the QM/MM calculations using the M06-2X hybrid functional (Zhao and Truhlar, 2008a,b) with the standard 6-31+G(d,p) basis set, to treat the QM subset of 76 atoms (see Figure S2). The rest of the system was described applying the AMBER and TIP3P force fields, as implemented in the fDYNAMO library (Field et al., 2000; Krzemińska et al., 2015). The position of all atoms above 20 Å from KAN was frozen. In order to explore proposed mechanisms, the potential energy surfaces (PESs) at AM1, semiempirical level combined with MM force fields were computed. Based on chosen structures, a micro-macro iteration optimization algorithm (Turner et al., 1999; Martí et al., 2005) at M06-2X/MM level was used to localize, optimize, and characterize the TS structures using a Hessian matrix containing all the coordinates of the QM subsystem, whereas the gradient norm of the remaining movable atoms was maintained at <0.01 kcal·mol^−1^·Å^−1^. Intrinsic reaction coordinates (IRCs) were traced down from located TSs to the valleys of the reactants and products in mass-weighted Cartesian coordinates. Subsequently, last structures from IRC were used to localize, optimize, and characterize the ground states, i.e., reactant and product complexes.

### Free Energy Calculations

To describe the mechanism of the reaction in condensed media, a free energy perturbation (FEP) (Świderek et al., 2013) method was used employing the M06-2X DFT functional to describe the QM sub-set of atoms. This method is usually second in choice, after umbrella sampling (Torrie and Valleau, 1977) (US), in terms of a potential mean force (PMF), and the selection is often dictated by a number of coordinates involved in approximate reaction coordinate (Świderek et al., 2015a). However, in these studies FEP employment originates in the limitations of semiempirical methods (SM) since, as it was shown recently by Otyepka and co-workers (Mlýnský et al., 2014) SM can easily fail and provide wrong conclusions about mechanistic paths in such theoretical models where the organic phosphorus atom is directly involved in chemical reactions.

Since FEP required the sampling of the environment along IRC traced previously from TS located at QM/MM level, the free energy pathway is obtained along a realistic reaction coordinate. Nevertheless, the limitation of this technique is assigned to lack of sampling on the chemical system, since just one characterized TS structure is used. Nevertheless, the FEP method opens the possibility of exploring the reaction path directly at a high level of theory. Thus, the QM wave function obtained at DFT level is polarized by the charges of the MM subset of atoms.

Free energy differences were estimated by mean of FEP methodology for the structures obtained along the IRC characterized by a single s coordinate:

(1) $$\begin{array}{l} s_{i}=s_{i-1}\\ \;+\sqrt{\underset{j\epsilon QM}{\sum }m_{j}\left({\left(x_{j,i}-x_{j,i-1}\right)}^{2}+{\left(y_{j,i}-y_{j,i-1}\right)}^{2}+{\left(z_{j,i}-z_{j,i-1}\right)}^{2}\right)} \end{array}$$

where *x*_*j, i*_ , *y*_*j, i*_, and *z*_*j, i*_ are the coordinates of the i_th_ structure for the j_th_ QM atom belonging to the IRC traced from the transition state structure (x_j0_, y_j0_, and zj_0_ coordinates) and *m*_*j*_ represents the corresponding masses of the atoms. Therefore, the free energy relative to the reactant is expressed as a function of the s coordinate as explained elsewhere (Świderek et al., 2013; Viciano et al., 2015). The MD simulations for the FEP calculation were performed at 300 K, using the NVT ensemble for the each window. 20 ps of production, with a time step of 1 fs, were completed. The total amount of windows required to generate the complete free energy path was 66 for the ATP-assisted, 37 for the Glu145-assisted mechanism in enzyme and 101 for reaction in water.

### Kinetic Isotope Effects

Kinetic isotope effects (KIEs) have been computed for isotopic substitutions of key atoms, from the TSs and the reactant complexes localized at the M06-2X/MM level of theory. Detailed information about the method used herein for computing KIEs can be found elsewhere (Świderek et al., 2014, 2017a).

## Results and Discussion

### Reaction Mechanism

**Figure 4:**
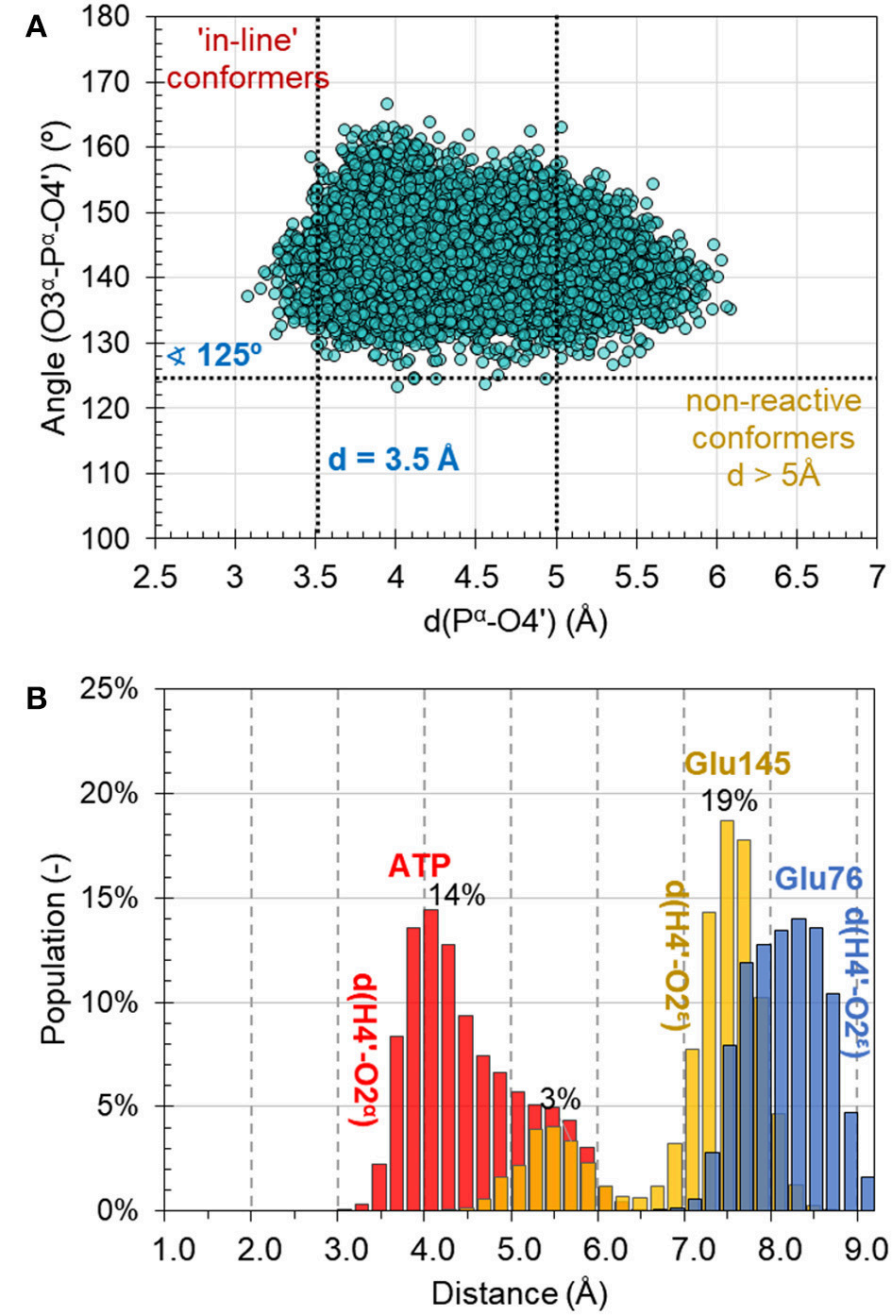
**(A)** Population of structures of reactant complex generated along the 10 ns of classical AMBER/TIP3P at NVT MD simulations as a function of the O3α-Pα-O4′ angle and the O4′-Pα distance. **(B)** Distribution of catalytically important distances along MD simulations.

MD simulations on the reactant complex at MM level shed some light onto the possible reaction mechanisms, to be later explored at QM/MM level. First, as can be seen in Figure 4A, the “in-line” position of the 4′-hydroxyl group of KAN and pyrophosphate of ATP required for ensuring direct attack at the α-phosphorus atom, exists in only 2% of cases of 10,000 overall structures saved along 10 ns of MD simulations [note: distance between O4′-Pα^(ATP)^ not longer than 3.5 Å, and angle not smaller than 125° for O3α-O4′-Pα^(ATP)^ were assigned as boundary conditions, similar to the definition used by York and co-workers (Heldenbrand et al., 2014)]. This result supports the statement that in-line conformations are often rare. Moreover, the free energy required to bring the nucleophile in-line has been predicted, at least in some cases, to be only modest and likely not a dominant factor on the overall catalytic rate (Min et al., 2007). In the X-ray structure, the same distance between nucleophile and Pα^(ATP)^ of ATP imitator, AMPCPP equal to 5.0 Å, was originally interpreted as corresponding to the inactive conformation. Based on analysis made for this distance it is observed that the change of AMPCPP to ATP reduces the presence of non-reactive conformations to 15% of the entire population of structures generated along the MD. Nevertheless, a rather large spread in the values of the distances and angles can still be observed, suggesting that both substrates are not completely immobilized in the binding pocket of ANT(4′).

Secondly, the analysis of the distribution of distances indicates that the O4′ atom of KAN, which plays the role of a nucleophile in the studied reaction, can be activated by either O2α^(ATP)^ of the ATP-cofactor, or OE2^(Glu145)^ from Glu145, and rather not by Glu76 in the wild-type variant of ANT(4′). According to the population analysis, in c.a. 32% of the snapshots, the strongest interaction between H4′ and O2α was found with distance lower than 4 Å, as shown in Figure 4B.

Glu145 approaches the OH4′ group at a distance lower than 5 Å only in 5% of cases. However, Glu76 is located much farther and not closer than 7 Å. These observations are especially interesting taking into account results of experimental kinetic studies done for a single Glu145Gln, or Glu76Gln, and double Glu76Gln/Glu145Gln mutated variants of ANT4′ (Matesanz et al., 2012). In these studies, a loss of catalytic activity was observed only in case of the double mutated enzyme, suggesting that ANT(4′) is equipped with duplicated basis catalyst. However, from results of MD obtained in this work it can be concluded that, in case of the wild-type variant, the main role of catalytic base is played by Glu145, but it is possible that its role is taken over by Glu76 once the Glu145 is absent in the active site.

**Figure 5:**
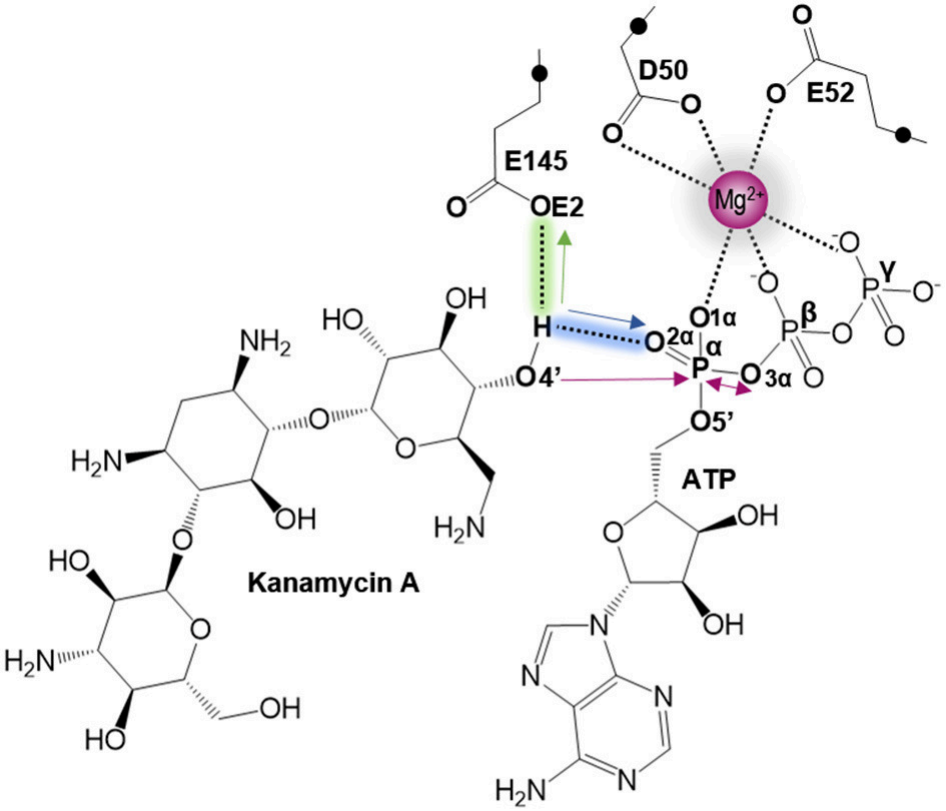
Schematic representation of the two proposed mechanisms of KAN inactivation by ANT(4'). The blue and green arrows represent the two different paths of O4′ activation by ATP (for ATP-assisted) and by Glu145 (for Glu145-assisted mechanism), respectively. Pink arrows indicate common process for both proposed mechanism of nucleophilic attack of O4′ to Pα and Pα–mO3α bond cleavage.

**Figure 6:**
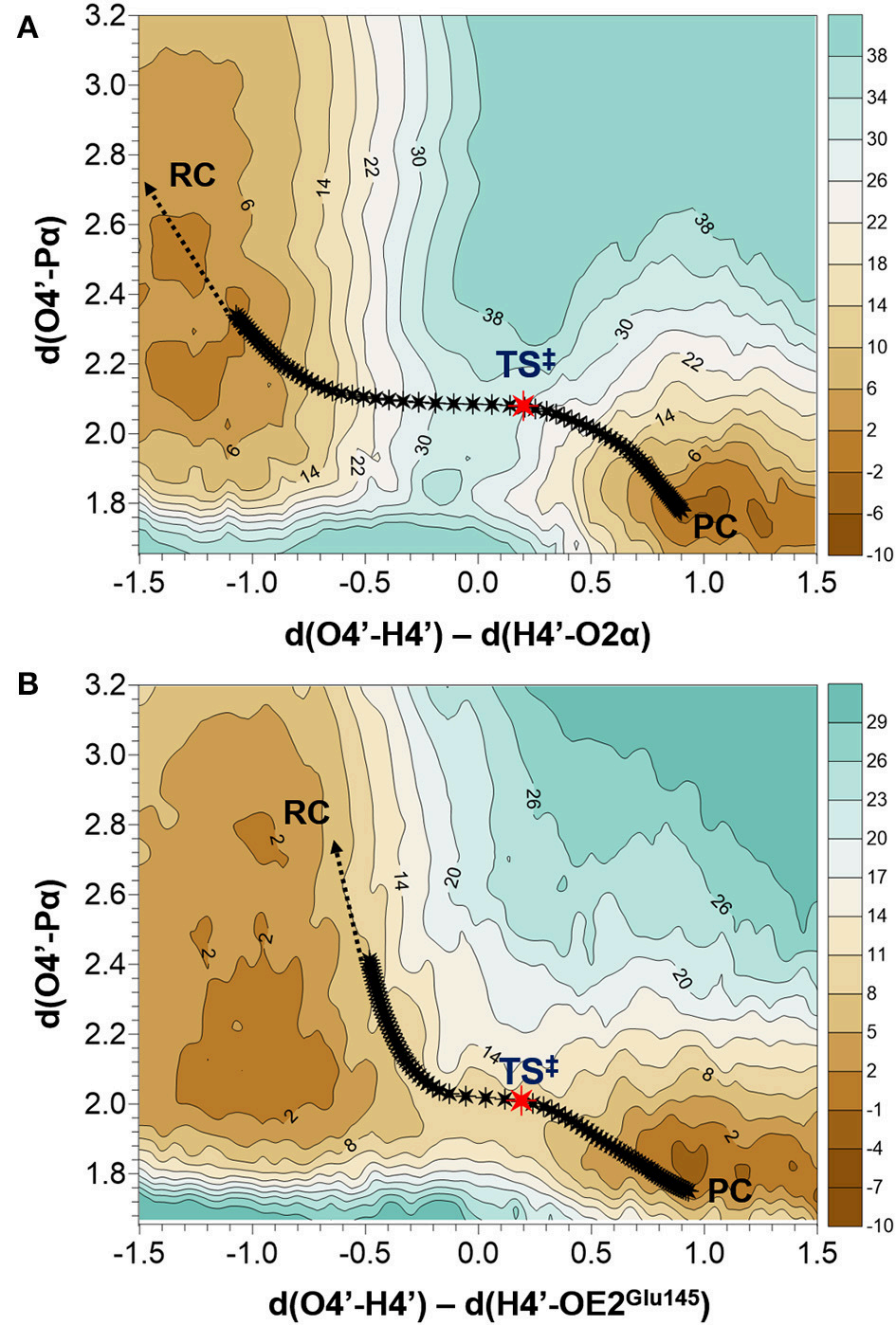
Potential energy surfaces obtained at AM1/MM level with superposition of located transition states and the projection of the reaction pathway obtained along IRC calculations obtained at M06-2X/MM level for **(A)** ATP-assisted and **(B)** Glu145-assuted mechanisms. Isoenergetic lines are given in kcal·mol^−1^ and distances in Å.

**Figure 7:**
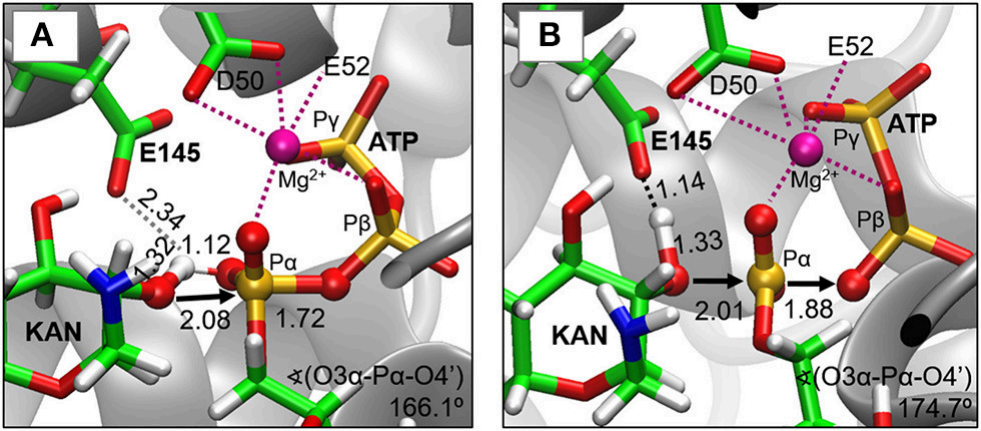
Structures of transition states localized at M06-2X/6-31+G(d,p)//AMBER/TIP3P level for **(A)** ATP-assisted and **(B)** Glu145-assisted mechanisms. Distances in Å.

As indicated by key interactions found along the MD trajectory, two possible mechanisms for the adenylyl group transfer should be considered, i.e., ATP-assisted and Glu145-assisted mechanism, as shown in Figure 5. In both proposed mechanisms, the common process of nucleophilic attack of O4′ to Pα is expected to take place together with the Pα-O3α bond cleavage, and the only difference is related to the origin of acceptor of transferred H4′ from O4′ of the hydroxyl group. In the case of the ATP-assisted mechanism, H4′ is transferred to O2α, the oxygen of phosphate group not involved in interaction with Mg^2+^ cation, while in the Glu145-assisted mechanism the same proton is transferred to the OE2 atom of the deprotonated carboxyl group of glutamic acid 145. Thus, in order to explore possible reaction pathways, two potential energy surfaces were computed at AM1/MM level (for details see Computational Method section), describing activation of O4′ of KAN by controlling antisymmetric combination of distances between the oxygen atom from the hydroxyl group of KAN (O4′) and its proton (H4′) and the same proton H4′ and its possible acceptor i.e., O2α or OE2^Glu145^, for ATP-assisted or Glu145-assisted mechanism, respectively, together with nucleophilic attack of O4′ to Pα of ATP. PESs are presented in Figure 6. The position of the TS and the reaction pathway traced by IRC calculation computed at the M06-2X/MM level are projected on the surfaces. As can be noticed, the position of the localized M06-2X/MM TS structures are quite close to quadratic regions defined on PES computed at AM1/MM level. Based on the shape of the obtained surfaces and after analysis of geometries of localized TS structures,(for which geometrical coordinates are given in Table S5 and structures are presented in Figure 7), it is confirmed that both reactions proceed *via* concerted mechanisms with only one TS formed along the chemical path. Structures of both optimized TSs have an associative character, [see (More O'Ferrall, 1970; Jencks, 1985) plot presented in Figure S3 and Table S3] in which nucleophilic attack slightly precedes the living group departure.

Additionally, the free energy barriers for both mechanisms were computed at M06-2X/MM level using the FEP method. The resulting activation free energies are 53.4 and 12.2 kcal·mol^−1^ for the ATP-assisted and Glu145-assisted mechanisms, respectively (the computed profiles are presented in Figure S4). These results clearly indicate that the second mechanism is the more favorable one. Moreover, a stable (of −1.6 kcal·mol^−1^) product complex is formed only in this mechanism. In contrary, in ATP-assisted mechanism the obtained product complex is energetically much more unstable, with energy of 40.3 kcal·mol^−1^ with respect to the reactant complex. Thus, it can be concluded that inactivation of KAN in the active site of ANT(4′) takes place with direct participation of the Glu145 residue in the chemical process.

It is worth mentioning that the computed free energy barrier of 12.2 kcal·mol^−1^ for the Glu145-assisted mechanism is much smaller than those deduced from experimentally measured rate constants of 19.7 kcal·mol^−1^ (for k_cat_ = 0.06 ± 0.01 measured in 35°C) (Revuelta et al., 2008), and 17.2 kcal·mol^−1^ (for k_cat_ = 1.3 ± 0.1 s^−1^ measured in 25°C) (Gerratana et al., 2001). This meaningful difference between experimental and theoretical values can be explained based on the fact that the rate-limiting step in this process is the release of the product rather than the chemical step, and thus experimental barriers can be treated, at most, as the upper limit value.

### Kinetic Isotope Effects (KIEs)

In order to ensure our predictions, the reaction mechanisms described above can now be examined using such a sensitive tool as heavy-atom kinetic isotope effects (KIEs). KIEs are defined as the ratio of rate constants for the reactions involving the light and the heavy isotopically substituted reactants, and reflect changes in bond order between the ground and rate-limiting transition states. In this section, the interpretation of KIEs obtained theoretically for studied reaction is done, including comparison with experimentally measured intrinsic KIEs for bridge and non-bridge oxygen atoms with labeled slow substrate analog *m*-nitrobenzyl triphosphate (*m*NBTP) instead of ATP (Gerratana et al., 2001). The change of the substrate seems not to influence the reaction mechanism, which still has the same regiospecificity, however it slows down inactivation of KAN of 2 orders of magnitude (Gerratana et al., 2001).

As shown in Table 1, in both ATP-assisted and Glu145-assisted mechanisms, normal primary KIEs (1°-KIE) of 0.9% and 1.2% for isotopically substituted oxygen in position 3α (oxygen of the leaving group), respectively, were computed. Both 1°-KIE values are in very good agreement with the experimentally measured value of 1.4% (Gerratana et al., 2001). In all cases, the existence of normal 1°-KIE reflects loss of bond order formed between phosphorus atom Pα and the bridge oxygen O3α in the TS with respect to reactant complex. This result can surely indicate that in the rate-limiting step, the P-O3α bond is cleaved. However, 1°-KIE values make it impossible to distinguish between ATP- and Glu145-assisted mechanisms and, moreover, to provide conclusive evidence if reaction proceed *via* concerted or step-wise mechanism. In such a case determination of 2°-KIE is crucial to dispel these doubts. Calculation of KIE for ^18^O-substituted non-bridge oxygen such as O5′, O1α, and O2α has been done. As can be deduced from the results presented in Table 1, the total 2°-KIE for the ATP-assisted mechanism is slightly inverse, ca. −1% in contrast to the KIE computed for the Glu145-assisted mechanism with a very small normal KIE of 0.07%. The last one indicates clearly that loss of bond order for the non-bridge oxygens occurs simultaneously with P-O3α bond breaking and reveals a concerted mechanism. Existence of the small normal 2°-KIE is also observed experimentally (Gerratana et al., 2001), supporting our previous conclusion based on energetic analysis that the Glu145-assisted mechanism is the most favorable candidate and should correspond to the most realistic reaction pathway. Interestingly, the inverse 2°-KIE observed in the case of the ATP-assisted mechanism can be explained by the protonation of the non-bridge oxygen, O2α. Nevertheless, this result is in complete disagreement with obtained experimental results, which allows us once again to discard the ATP-assisted mechanism.

**Table 1:** Primary and secondary kinetic isotope effects computed for isotopically substituted bridge and non-bridge oxygen atoms of ATP, respectively, in the ATP-assisted and Glu145-assited mechanism of KAN inactivation by ANT(4′).

	Exp.*	**Atoms**	**ATP-assisted**	**Glu145-assisted**
1°-KIE ^18^O-bridge (O3α)	1.014, 0.002	O3α	1.0092	1.0121
2°-KIE^18^O-non-bridge (O1α, O2α, O5')		O5'	1.0041	1.0026
	1.0024, 0.0002	O1α	0.9980	1.0000
		O2α	0.9884	0.9981

* *Provided experimental values correspond to KIEs measured at pH = 7.7, labeled slow substrate analog m-nitrobenzyl triphosphate (mNBTP) and are taken from (Gerratana et al., 2001)*.

### Electrostatic Effects

The most accepted hypothesis explaining the origin of the enzymatic catalysis is based on an assumption that stabilization of TS is achieved by means of better electrostatic interactions with the protein compared with its equivalent reaction in solution (Adamczyk et al., 2011; Moliner, 2011). Validity of this hypothesis was examined and strongly supported by our previous results obtained for systems such as protease HIV-1 (Krzemińska et al., 2016), glycine N-methyltransferase (GNMT) (Świderek et al., 2018) and *de novo* designed KEMP eliminase (Świderek et al., 2015b, 2017b). Thus, we decided to test this hypothesis also in the case of ANT(4′). For this purpose, one additional theoretical model was built, where KAN together with MgATP was solvated in a box of explicit water molecules. Additionally, in order to obtain the same reaction mechanism as proposed for Glu145-assited pathway, the presence of a base was required, and this was fulfilled by adding the propionate molecule as imitator of Glu residue to the model. Afterwards, TS structure was localized at M06-2X/MM level (its geometrical coordinates are given in Table S6). Subsequently, identical procedure, as explained in the computational method section was applied in order to explore free energy surface. Free energy surface obtained for reaction in aqueous solution is shown in Figure S5. As it is well-known, non-catalyzed phosphoryl transfer reactions are extremely slow, and enzymes can provide rate enhancements of >10^20^-fold (Lad et al., 2003).

Hence, our target herein was to understand the origin of enzyme catalytic power. In other words, the role of the enzyme in the KAN deactivation process is explored based on analysis of electrostatic potential generated by protein in a key position, i.e., on Pα atom, V^Pα^(r) the center of transferred group. Since the rate-limiting step of the reaction catalyzed by ANT(4′) is the release of the KAN-AMP complex from the active site, it can be assumed that this enzyme achieved the highest possible catalytic effect to enhance the speed of this reaction.

The obtained results of V^Pα^(r), generated by ANT(4′) and water solvent together with formal charge accumulated on the transferred group, are collected in Table 2 and Table S4. A comparison of these magnitudes computed for TS structures for the reaction occurring through the base-assisted mechanism in enzyme and aqueous solution reveals the existence of a much larger positive V^Pα^(r) generated on the negatively charged transferred group in the active site of the enzyme (801.5 ± 17.7 kJ·mol^−1^) than in the water (628.2 ± 49.9 kJ·mol^−1^). According to the basic laws of physics, larger positive electrostatic potential should better stabilize the transition state in which accumulation of strong negative charge of −0.698 and −0.619 a.u., in water and enzyme, respectively, is observed in this atom. Therefore, as a result of this stabilization, the reduction of the free energy barrier in enzyme is expected. This expectation is fulfilled by theoretical results where a higher barrier (of 47.9 kcal·mol^−1^) was computed for reactions taking place in water solvent than in enzyme (12.2 kcal·mol^−1^). Thus, the reduction of the rate constant of ca. 10^26^-fold can be considered, according to presented hypothesis, to arise from the stronger electrostatic potential created by ANT(4′). The possible contribution of each amino acid residue to the catalytic power was later explored, indicating their specific role in catalysis. It was found that the highest contribution to the overall positive value of the electrostatic potential comes from the positively charged residues located in the surroundings of the active site such as Lys-149 (~33.7%) from chain A, and Arg-42 (~13.5%) and Lys-74 (~10.7%) from chain B (see Figure S7). These residues can be considered to have the highest impact on enhancing the rate constant of the chemical reaction. On the other hand, nearby negatively charged Glu-141 and Glu-142 from chain A and Glu-76 from chain B produce negative electrostatic potential. Nevertheless, this unfavorable effect is too small to perturb the overall potential magnitude.

**Table 2:** Atomic charge (in a.u.) computed for structures localized at M06-2X/AMBER/TIP3P level of theory using the ChelpG method assigned to the transferred -PαO3R group, with electrostatic potential (in kJ·mol^−1^) generated by ANT(4′) in Pα position in reactant complex and transition state, donor-acceptor distance (Å) and free energy barriers (ΔG^‡^ in kcal·mol^−1^).

**Mechanism**		**Charge^a^ (a.u)**	**V^***Pα***^(r) (kJ·mol^**−1**^)**	**DAD (Å)**	**ΔG^**‡**^ (kcal·mol^**−1**^)**
ATP-assisted	RC	−0.846^a^	834.0 ± 17.5	4.45	53.4
	TS	−0.130^b^	747.1 ± 20.4	3.77
Glu145-assisted	RC	−0.582^a^	824.6 ± 24.4	4.26	12.2
	TS	−0.619^a^	801.5 ± 17.7	3.88
Water	RC	−0.734^a^	586.3 ± 38.8	4.30	47.9
	TS	−0.698^a^	628.2 ± 49.9	3.81

a presented values of charges correspond to sum of formal charges computed for Pα, O1α, O2α, O5' atoms

b *in case of TS for ATP-assisted the charge of transferred H4' to O2α was added*.

Finally, the analysis done for ATP-assisted and Glu145-assisted mechanisms occurring in the same active site shows, as expected, that values of V^Pα^(r) generated on the transferred group in TS and RC are very similar. However, the meaningful difference is found in the charge distribution on substrates. (see Table 2) For the most favorable mechanism (with lower free energy barrier), a slight increase of negative charge on the phosphate group in TS in respect to RC (−0.619 and −0.582 a.u., respectively) is observed. However, the largest change in charge distribution from RC to TS was found in the ATP-assisted mechanism. In this case the larger negative charge of −0.846 a.u. is observed in RC. Since the H4′ proton is transferred from the hydroxyl group of KAN to the adenylyl group in this mechanism, its presence dramatically decreases the negative charge accumulated on the adenylyl group up to −0.130 a.u in TS. Consequently, V^Pα^(r) generated by ANT(4′) in the case of the ATP-assisted mechanism would rather over-stabilize the ground state in comparison to the TS structure, and this could explain the high free energy barrier obtained for this mechanism.

### Compression Effects

**Figure 8:**
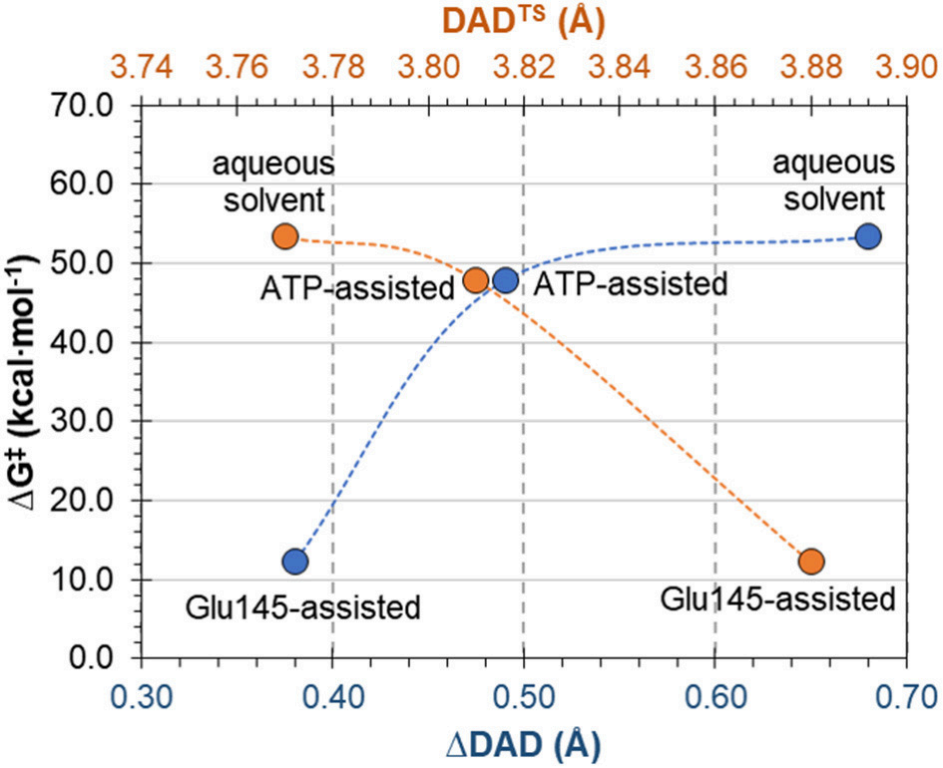
Free energy barrier ΔG^‡^ for reaction occurring in enzyme and water vs. donor-acceptor distance in transition state, DAD^TS^ and its change from reactants to transition state ΔDAD^(RC−TS)^.

The complementary hypothesis for enzymes catalyzing S_N_2 reactions is the so-called “compression hypothesis,” originally proposed by Showen (Hegazi et al., 1979) for enzymatic methyl transfer. Extrapolated to hydrogen transfer reactions (Roston et al., 2013), this hypothesis explains the origin of the enzymatic catalysis, suggesting that specific protein fluctuations might reduce the donor-acceptor distance (DAD), and as a consequence decrease the reaction barrier by increasing the number of reactive trajectories (Kohen, 2015). Thus, since mechanistic results of the transfer of the adenylyl group presented in this work have revealed its S_N_2 character, we decided to use this reaction as an example that could shed some new light and contribute to the ongoing debate about the validity of the proposed hypothesis. For this purpose, the evolution of DAD vs. distances describing the –PO_3_R group transfer was analyzed for aqueous and enzymatic reaction, as presented in Figure S6. As it was observed, the decrease of the DAD from RC to TS is 0.68 and 0.38 Å for the ATP and Glu145-assisted mechanisms, respectively. DAD values are provided in Table 2. Interestingly, the minimum value of DAD (DAD_min_) is not achieved in TS, and in both cases it appears much beyond the highest energetic point along the reaction pathway, to be then elongated to allow for product formation. Decrease of a DAD is also observed in reaction in water, where ΔDAD^(RC−TS)^ was found to be 0.49 Å. However, in contrast to enzymatic reactions, in this case the DAD value decreases constantly until a pentavalent intermediate is formed. Analysis of the influence of DAD distance in TS to the values of the free energy barriers, as plotted in Figure 8, reveals that short DAD^TS^ does not guarantee reduction in the reaction barrier. Nevertheless, an interesting relation was found indicating that the larger change in DAD from RC to TS, the slower the reaction is. This would suggest that large changes in DAD require more energy to be involved in the chemical process. Finally, it can be concluded that reduction of the reaction barrier is achieved only when the short DAD in the Michaelis complex is reached, i.e., 4.26 Å for the Glu145-assisted mechanism, vs. 4.45 Å for the ATP-assisted mechanism or 4.30 Å for reactions taking place in water.

## Conclusions

In this work the molecular mechanism of the transfer of the adenylyl group from ATP to KAN, and formation of KAN-AMP-Mg ternary complex as a result of the reaction catalyzed by the ANT(4′) enzyme was investigated. Two proposed mechanisms were studied, the ATP-assisted and Glu145-assited mechanisms, which were shown to proceed in a concerted manner, in which the O4′ atom of KAN is activated by proton abstraction by either ATP or Glu-145 residue, respectively, and at the same time formation of the O4′-Pα bond and breaking of the Pα-O3α bond occur. Based on the obtained free energy barriers, it was deduced that the most favorable mechanism for KAN deactivation by ANT(4′) proceeds *via* the Glu145-assisted mechanism, with the barrier of 12.2 kcal·mol^−1^ much smaller than that obtained for the ATP/Mg-assisted process (53.4 kcal·mol^−1^). This value is slightly underestimated when compared to the barriers obtained from experimentally measured rate constants, i.e., between 17.2 and 19.7 kcal·mol^−1^. The lower barrier obtained in theoretical studies can be explained by the fact that the rate-limiting step in this reaction is, in fact, not the chemical conversion, but the release of the KAN-AMP complex. Then, the theoretical prediction of the most favorable mechanism was confirmed by determination of KIEs. This sensitive tool allowed us to discriminate between both mechanisms based on 2°-KIEs computed for isotopic substitution of non-bridge oxygen atoms. In the case of the ATP-assisted mechanism, the inverse (<1) value of 2°-KIE was obtained, being completely opposite to the experimentally measured data. However, very good agreement between the experimentally and theoretically determined primary and secondary KIEs was found in the case of the Glu145-assisted mechanism, confirming its existence.

Additionally, calculation of the electrostatic potential generated by enzymes on atoms involved in chemical reactions reveals that electrostatic effects can be correlated with the height of computed free energy barriers. Thus, the high barrier of 47.9 kcal·mol^−1^ observed for the reaction in the water solvent can be explained by the low positive potential (of c.a. 628.2 ± 49.9 kJ·mol^−1^) generated on the negatively charged adenyl-group. In contrast, the higher electrostatic potential of ca. 800 kJ·mol^−1^ generated in the active site of the enzyme stabilizes a much better structure of TS and as a result reduces the reaction barrier ca. 4-fold.

Moreover, in the case of reactions with more than one possible mechanism as the one under study in the present paper, the obtained results allow us to conclude the important role of enzymes to direct chemical transformation through the reaction mechanism in which distribution of charges on key atoms in the structures of TS is the most compatible one to its electrostatic potential.

Finally, the “compression hypothesis” for reaction of S_N_2 character was examined, indicating that a short donor-acceptor distance in TS does not guarantee reduction in the reaction barrier, and that this reduction is achieved only when the short DAD in the Michaelis complex is found.

## Author Contributions

SM implemented FEP method in fDynamo library and computed free energy surfaces. AB served as an expert in ANT(4′) enzyme, corrected the manuscript. KŚ designed the studies, ran calculations, analyzed results and finally wrote the manuscript.

### Conflict of Interest Statement

The authors declare that the research was conducted in the absence of any commercial or financial relationships that could be construed as a potential conflict of interest.

## Footnotes

^1^Center of Disease Control and Prevention. Available online at: https://www.cdc.gov/drugresistance/biggest_threats.html

## Supplementary Material

Supplementary Table 1 Empirically predicted pKa values for titratable residues. Atom types, charges and parameters obtained for Kanamycin A used in MM simulations. Results of MD simulations: RMSD, RMSF, evolution of energy and thermostat control. Schematic representation of active site. O'Ferrall-Jencks plot for ATP-assisted and Glu145-assisted mechanism. Free energy profiles for ATP-assisted and Glu145-assisted mechanism catalyzed by ANT(4′) computed at M06-2X/6-31+G(d,p)//AMBER/TIP3P level. Free energy profile for base-assisted mechanism in aqueous solution computed at M06-2X/6-31+G(d,p)//AMBER/TIP3P level. Key distances and angles for reactant complex, transition state and product complex localized along ATP-assisted and Glu145-assisted mechanism at M06-2X/6-31+G(d,p)//AMBER/TIP3P level. Atomic charge computed for structures localized at M06-2X/AMBER/TIP3P level. Geometrical coordinates of QM atoms for transition state structures localized at M06-2X/6-31+G(d,p)//AMBER/TIP3P level for reaction catalyzed by ANT(4′). Evolution of donor-acceptor distance (DAD) along reaction path in catalyzed and uncatalyzed reaction. Contribution of key amino acid residues to overall value of electrostatic potential. Geometrical coordinates of QM atoms for transition state structure localized at M06-2X/6-31+G(d,p)//AMBER/TIP3P for reaction is aqueous solution.
