# Supplementary material for: Theoretical Studies on Mechanism of Inactivation of Kanamycin A by 4′-O-Nucleotidyltransferase
Source: Front Chem. 2019 Jan 29;6:660. doi: 10.3389/fchem.2018.00660 (PMC6361787; doi:10.3389/fchem.2018.00660)
Supplement: Supplementary Table 1 — Empirically predicted pKa values for titratable residues. Atom types, charges and parameters obtained for Kanamycin A used in MM simulations. Results of MD simulations: RMSD, RMSF, evolution of energy and thermostat control. Schematic representation of active site. O'Ferrall-Jencks plot for ATP-assisted and Glu145-assisted mechanism. Free energy profiles for ATP-assisted and Glu145-assisted mechanism catalyzed by ANT(4′) computed at M06-2X/6-31+G(d,p)//AMBER/TIP3P level. Free energy profile for base-assisted mechanism in aqueous solution computed at M06-2X/6-31+G(d,p)//AMBER/TIP3P level. Key distances and angles for reactant complex, transition state and product complex localized along ATP-assisted and Glu145-assisted mechanism at M06-2X/6-31+G(d,p)//AMBER/TIP3P level. Atomic charge computed for structures localized at M06-2X/AMBER/TIP3P level. Geometrical coordinates of QM atoms for transition state structures localized at M06-2X/6-31+G(d,p)//AMBER/TIP3P level for reaction catalyzed by ANT(4′). Evolution of donor-acceptor distance (DAD) along reaction path in catalyzed and uncatalyzed reaction. Contribution of key amino acid residues to overall value of electrostatic potential. Geometrical coordinates of QM atoms for transition state structure localized at M06-2X/6-31+G(d,p)//AMBER/TIP3P for reaction is aqueous solution. [file Table_1.DOCX]

Supplementary Material

Theoretical Studies on Mechanism of Inactivation of Kanamycin A by 4’-O-Nucleotidyltransferase.

Sergio Marti,^1^ Agatha Bastida,^2^ Katarzyna Świderek^1,^*

^1^Department de Química Física i Analítica, Universitat Jaume I, 12071 Castelló, Spain
^2^Departamento de Química Bio-orgánica, Instituto de Química Orgánica General (CSIC), 28006 Madrid, Spain.

*** Correspondence:** Katarzyna Świderek: swiderek@uji.es

**Table S1.** Empirically predicted pKa values for titratable residues.

| Residue | pKa^calc.^ | pKa-AA | Residue | pKa^calc.^ | pKa-AA | Residue | pKa^calc.^ | pKa-AA |
| --- | --- | --- | --- | --- | --- | --- | --- | --- |
| Asp25 | 3.93 | 3.8 | Glu10 | 3.34 | 4.5 | Tyr27 | 10.16 | 10 |
| Asp29 | 3.9 | 3.8 | Glu11 | 4.2 | 4.5 | Tyr37 | 17.71 | 10 |
| Asp30 | 4.16 | 3.8 | Glu18 | 3.65 | 4.5 | Tyr48 | 10.09 | 10 |
| Asp45 | 2.83 | 3.8 | Glu21 | 4.68 | 4.5 | Tyr80 | 13.88 | 10 |
| Asp50 | -0.18 | 3.8 | Glu52 | 9.62 | 4.5 | Tyr88 | 12.23 | 10 |
| Asp87 | 4.01 | 3.8 | Glu60 | 4.58 | 4.5 | Tyr110 | 10.54 | 10 |
| Asp95 | 6.3 | 3.8 | Glu61 | 4.59 | 4.5 | Tyr115 | 9.91 | 10 |
| Asp111 | 3.13 | 3.8 | Glu63 | 4.91 | 4.5 | Tyr120 | 10.19 | 10 |
| Asp133 | 3.3 | 3.8 | Glu67 | 11.4 | 4.5 | Tyr146 | 14.71 | 10 |
| Asp200 | 2.23 | 3.8 | Glu72 | 4.78 | 4.5 | Tyr185 | 13.67 | 10 |
| Asp206 | 4.09 | 3.8 | Glu76 | 8.35 | 4.5 | Tyr 205 | 17.46 | 10 |
| Asp219 | 3.27 | 3.8 | Glu82 | 3.07 | 4.5 | Tyr243 | 11.89 | 10 |
| Asp246 | 3.18 | 3.8 | Glu83 | 5.03 | 4.5 | Arg9 | 13.45 | 12.5 |
| His17 | 6.05 | 6.5 | Glu93 | 5.36 | 4.5 | Arg12 | 11.03 | 12.5 |
| His66 | 4.92 | 6.5 | Glu117 | 4.74 | 4.5 | Arg22 | 13.76 | 12.5 |
| His100 | 4.22 | 6.5 | Glu127 | 4.6 | 4.5 | Arg42 | 9.25 | 12.5 |
| His132 | 6.77 | 6.5 | Glu141 | 6.39 | 4.5 | Arg151 | 12.23 | 12.5 |
| His180 | 5.08 | 6.5 | Glu142 | 8.86 | 4.5 | Arg154 | 13.03 | 12.5 |
| His181 | 5.71 | 6.5 | Glu145 | 7.06 | 4.5 | Arg182 | 12.1 | 12.5 |
| His207 | 6.16 | 6.5 | Glu194 | 5.03 | 4.5 | Arg240 | 12.45 | 12.5 |
| His241 | 6.0 | 6.5 | Glu221 | 4.83 | 4.5 | Arg250 | 12.95 | 12.5 |
| Cys55 | 12.85 | 9 | Glu225 | 4.58 | 4.5 | Lys14 | 10.44 | 10.5 |
| Cys136 | 10.81 | 9 | Glu228 | 3.32 | 4.5 | Lys20 | 10.27 | 10.5 |
| Cys184 | 12.78 | 9 | Glu236 | 4.52 | 4.5 | Lys26 | 11.54 | 10.5 |
| Cys209 | 11.92 | 9 | Glu239 | 4.57 | 4.5 | Lys32 | 10.92 | 10.5 |
|  |  |  |  |  |  | Lys74 | 14.09 | 10.5 |
|  |  |  |  |  |  | Lys118 | 11.54 | 10.5 |
|  |  |  |  |  |  | Lys124 | 10.59 | 10.5 |
|  |  |  |  |  |  | Lys149 | 8.6 | 10.5 |
|  |  |  |  |  |  | Lys197 | 10.41 | 10.5 |
|  |  |  |  |  |  | Lys222 | 10.63 | 10.5 |
|  |  |  |  |  |  | Lys249 | 10.58 | 10.5 |

**Table S2.** Atom types, charges and parameters obtained for Kanamycin A generated using GAFF antenchamber package included in AmberTools and used in MM simulations.

| Atom number | Atom Name | Atom Type | Charge | Atom number | Atom Name | Atom Type | Charge |
| --- | --- | --- | --- | --- | --- | --- | --- |
| 1 | C1 | c3 | 0.2959 | 36 | H3 | h1 | 0.0477 |
| 2 | C2 | c3 | 0.0901 | 37 | H4 | h1 | 0.0997 |
| 3 | C3 | c3 | 0.0821 | 38 | H5 | h1 | 0.0757 |
| 4 | C4 | c3 | 0.1231 | 39 | H6 | h1 | 0.0637 |
| 5 | C5 | c3 | 0.0601 | 40 | H7 | h1 | 0.0527 |
| 6 | C6 | c3 | 0.1698 | 41 | H8 | h1 | 0.0787 |
| 7 | C7 | c3 | 0.1545 | 42 | H9 | h1 | 0.0627 |
| 8 | C8 | c3 | 0.1231 | 43 | H10 | h1 | 0.0757 |
| 9 | C9 | c3 | 0.1071 | 44 | H11 | h1 | 0.0877 |
| 10 | C10 | c3 | 0.1171 | 45 | H12 | h1 | 0.0687 |
| 11 | C11 | c3 | 0.1415 | 46 | H13 | hc | 0.0447 |
| 12 | C12 | c3 | -0.1174 | 47 | H14 | hc | 0.0687 |
| 13 | C13 | c3 | 0.3019 | 48 | H15 | h2 | 0.0927 |
| 14 | C14 | c3 | 0.0931 | 49 | H16 | h1 | 0.0877 |
| 15 | C15 | c3 | 0.1035 | 50 | H17 | h1 | 0.0757 |
| 16 | C16 | c3 | 0.1171 | 51 | H18 | h1 | 0.0957 |
| 17 | C17 | c3 | 0.0711 | 52 | H19 | h1 | 0.0527 |
| 18 | C18 | c3 | 0.1364 | 53 | H20 | h1 | 0.0797 |
| 19 | N1 | n3 | -0.9028 | 54 | H21 | h1 | 0.0487 |
| 20 | N2 | n3 | -0.8978 | 55 | H22 | hn | 0.3698 |
| 21 | N3 | n3 | -0.9068 | 56 | H23 | hn | 0.3448 |
| 22 | N4 | n3 | -0.8958 | 57 | H24 | hn | 0.3488 |
| 23 | O5 | os | -0.4256 | 58 | H25 | hn | 0.3638 |
| 24 | O6 | oh | -0.5938 | 59 | H26 | hn | 0.3488 |
| 25 | O7 | oh | -0.5828 | 60 | H27 | hn | 0.3608 |
| 26 | O8 | oh | -0.5838 | 61 | H28 | hn | 0.3538 |
| 27 | O9 | os | -0.4506 | 62 | H29 | hn | 0.3678 |
| 28 | O10 | oh | -0.6048 | 63 | H30 | ho | 0.414 |
| 29 | O11 | os | -0.4516 | 64 | H31 | ho | 0.415 |
| 30 | O12 | os | -0.4346 | 65 | H32 | ho | 0.405 |
| 31 | O13 | oh | -0.5928 | 66 | H33 | ho | 0.424 |
| 32 | O14 | oh | -0.5928 | 67 | H34 | ho | 0.433 |
| 33 | O15 | oh | -0.5948 | 68 | H35 | ho | 0.418 |
| 34 | H1 | h2 | 0.0937 | 69 | H36 | ho | 0.418 |
| 35 | H2 | h1 | 0.1077 |  |  |  |  |
| GAFF Parameters: | | | | | | | |
| **BOND**  oh-ho 369.60 0.974  oh-c3 314.10 1.426  c3-h1 335.90 1.093  c3-c3 303.10 1.535  c3-os 301.50 1.439  c3-n3 320.60 1.470  n3-hn 394.10 1.018  c3-h2 326.40 1.100  c3-hc 337.30 1.092  **ANGLE**  oh-c3-h1 50.970 109.880  oh-c3-c3 67.720 109.430  ho-oh-c3 47.090 108.160  c3-c3-os 67.780 108.420  c3-c3-h1 46.360 110.070  c3-c3-c3 63.210 110.630  h1-c3-h1 39.180 109.550  c3-os-c3 62.390 112.450  os-c3-h1 50.840 108.820  os-c3-h2 50.840 108.580  os-c3-os 71.720 110.240  c3-c3-n3 66.180 110.380  c3-n3-hn 47.130 109.920  n3-c3-h1 49.390 109.920  hn-n3-hn 41.300 107.130  c3-c3-h2 46.020 111.590  c3-c3-hc 46.370 110.050  hc-c3-hc 39.430 108.350  **DIHEDRALS**  oh-c3-c3-os 1 0.144 0.000 -3.000  oh-c3-c3-os 1 1.175 0.000 2.000  oh-c3-c3-h1 1 0.000 0.000 -3.000  oh-c3-c3-h1 1 0.250 0.000 1.000  oh-c3-c3-c3 1 0.156 0.000 3.000  ho-oh-c3-h1 1 0.167 0.000 3.000  ho-oh-c3-c3 1 0.160 0.000 -3.000  ho-oh-c3-c3 1 0.250 0.000 1.000  c3-c3-os-c3 1 0.383 0.000 -3.000 | | | | c3-c3-os-c3 1 0.100 180.000 2.000  c3-c3-c3-h1 1 0.156 0.000 3.000  c3-c3-c3-c3 1 0.180 0.000 -3.000  c3-c3-c3-c3 1 0.250 180.000 -2.000  c3-c3-c3-c3 1 0.200 180.000 1.000  h1-c3-c3-os 1 0.000 0.000 -3.000  h1-c3-c3-os 1 0.250 0.000 1.000  h1-c3-c3-h1 1 0.156 0.000 3.000  c3-os-c3-h2 1 0.383 0.000 3.000  c3-os-c3-os 1 0.100 0.000 -3.000  c3-os-c3-os 1 0.850 180.000 -2.000  c3-os-c3-os 1 1.350 180.000 1.000  c3-c3-c3-n3 1 0.156 0.000 3.000  os-c3-c3-c3 1 0.156 0.000 3.000  h1-c3-os-c3 1 0.383 0.000 3.000  c3-c3-n3-hn 1 0.300 0.000 3.000  oh-c3-c3-n3 1 0.156 0.000 3.000  h1-c3-c3-n3 1 0.156 0.000 3.000  c3-c3-c3-h2 1 0.156 0.000 3.000  hn-n3-c3-h1 1 0.300 0.000 3.000  oh-c3-c3-h2 1 0.156 0.000 3.000  h1-c3-c3-h2 1 0.156 0.000 3.000  os-c3-c3-n3 1 0.156 0.000 3.000  c3-c3-c3-hc 1 0.160 0.000 3.000  n3-c3-c3-hc 1 0.156 0.000 3.000  h1-c3-c3-hc 1 0.156 0.000 3.000  oh-c3-c3-oh 1 0.144 0.000 -3.000  oh-c3-c3-oh 1 1.175 0.000 2.000  **NONBON**  oh 1.7210 0.2104  ho 0.0000 0.0000  c3 1.9080 0.1094  h1 1.3870 0.0157  os 1.6837 0.1700  n3 1.8240 0.1700  hn 0.6000 0.0157  h2 1.2870 0.0157  hc 1.4870 0.0157 | | | |

**
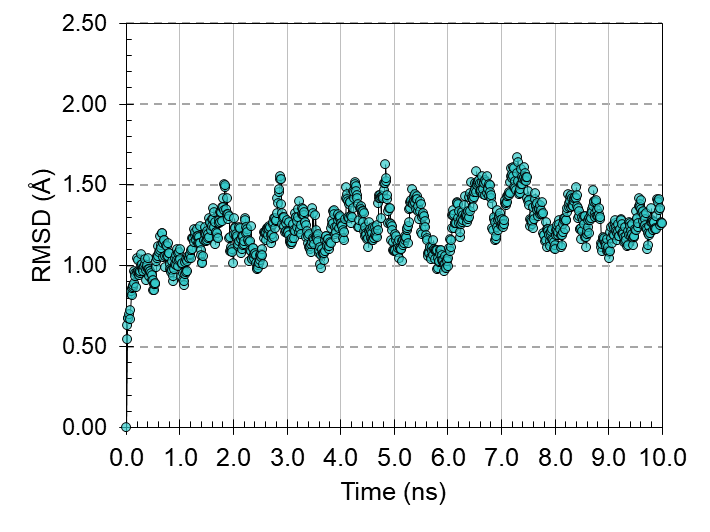

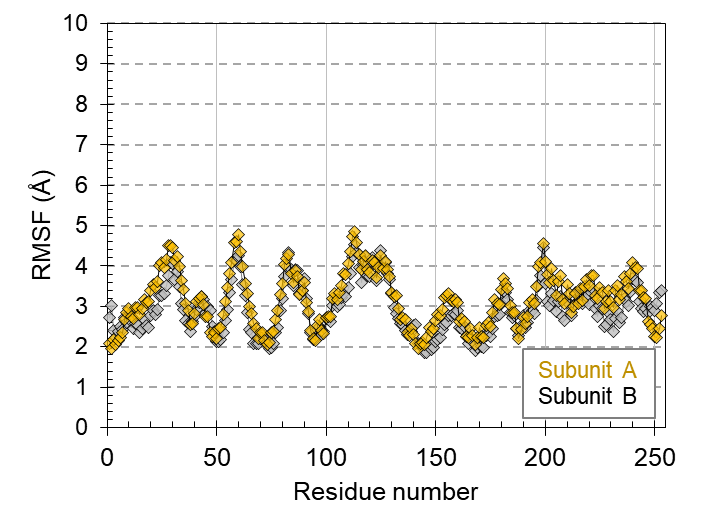
**

**
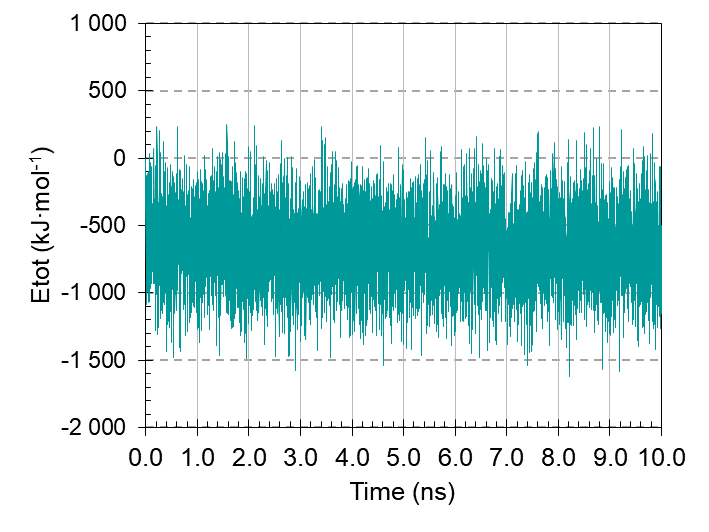

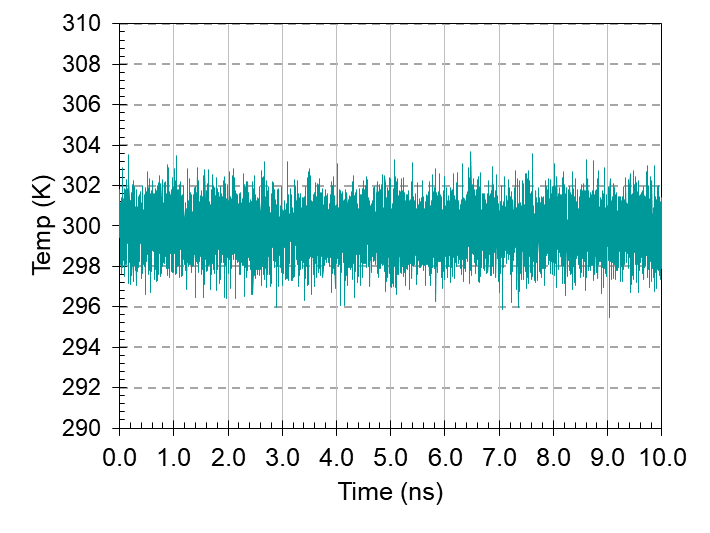

Figure S1.** **A.** Root mean square deviation (RMSD) of backbone (C-Cα-N) atoms, **B.** root mean square fluctuation (RMSF) of Cα-residues, **C.** Evolution of energy along MD simulations, **D.** Control of temperature.

**
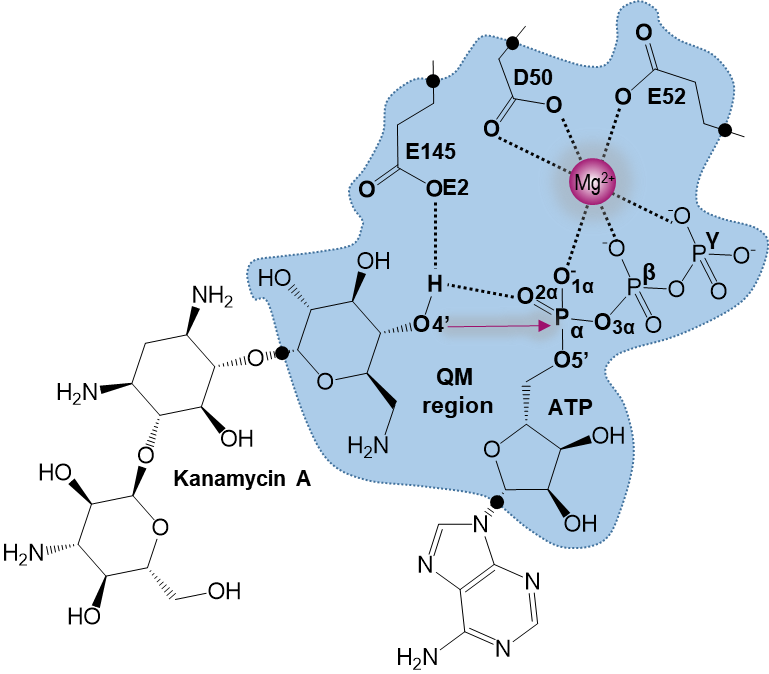
**

**Figure S2.** Schematic representation of active site with QM region distinguished by blue color. Five link atoms on the border between QM and MM region are indicated as black dots.


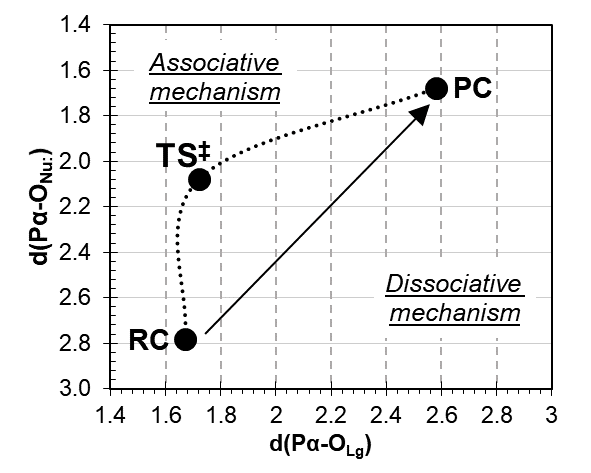

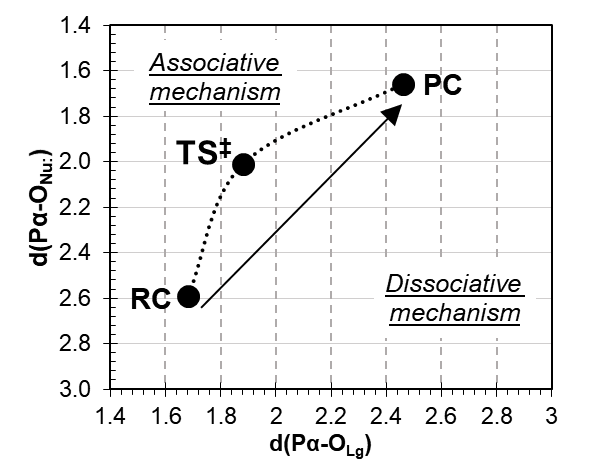


**Figure S3.** Positions on O’Ferrall-Jencks plot of localized TS structures at M06-2X/6-31+G(d,p) level for **A.** MgATP-assisted and **B.** Glu145-assisted mechanism.

**
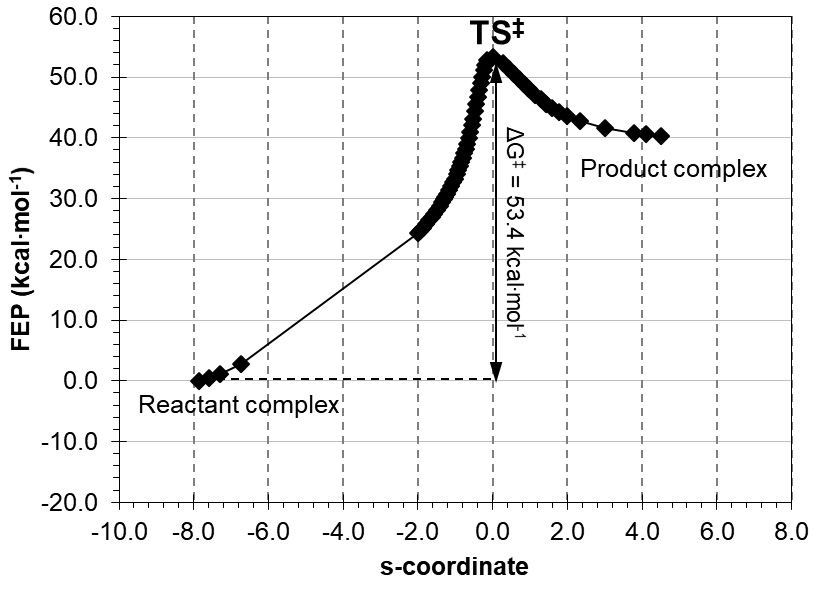

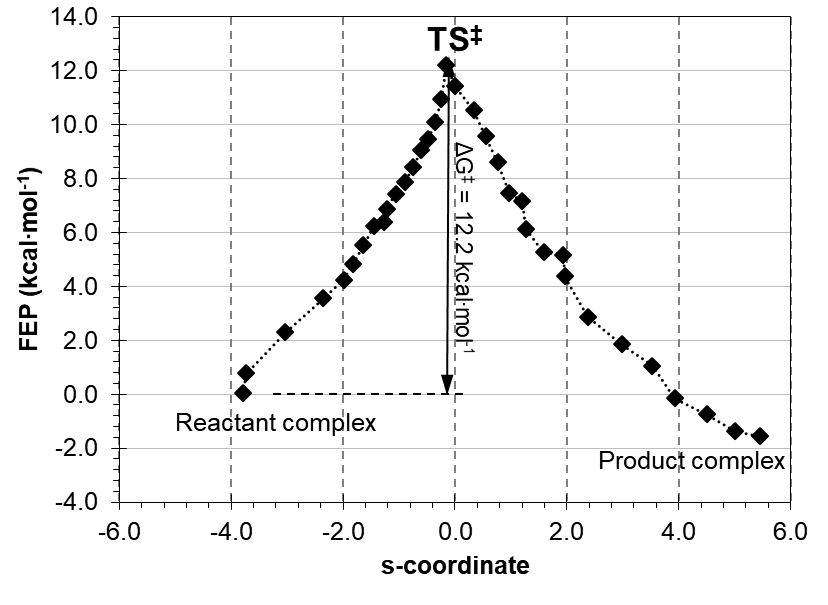
**

**
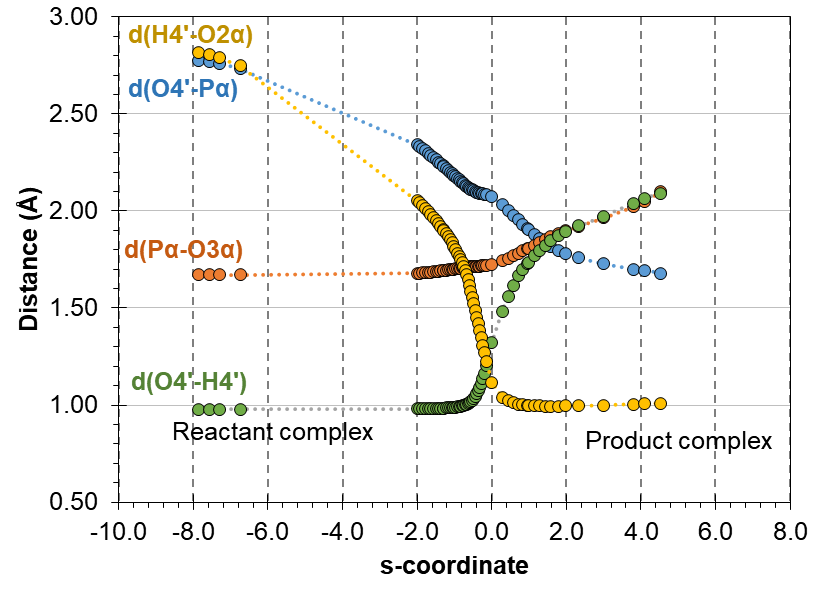

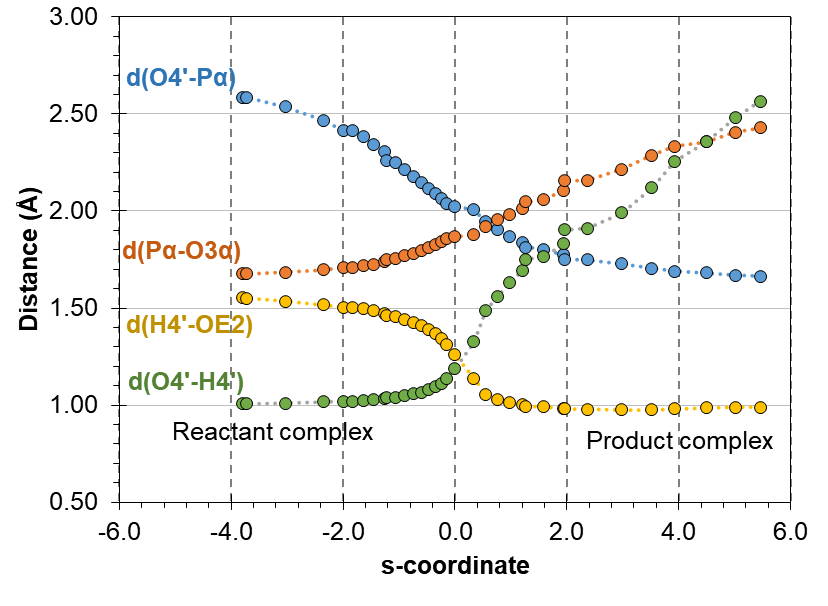
**

**Figure S4.** Free energy profiles for ATP-assisted (on left) and Glu145-assisted (on right) mechanism catalyzed by ANT(4’) computed using free energy perturbation method at M06-2X/6-31+G(d,p)//AMBER/TIP3P level of theory, together with evolution of key distances along reaction path.

**
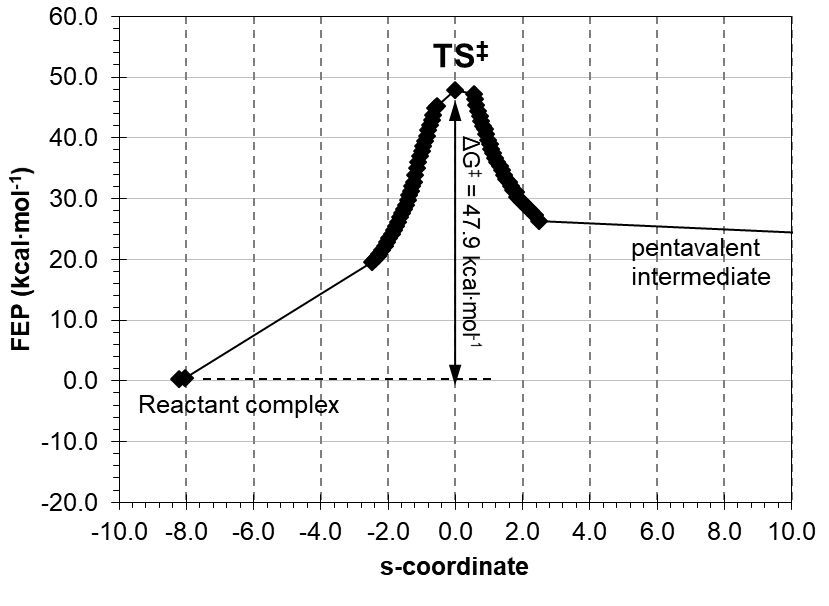

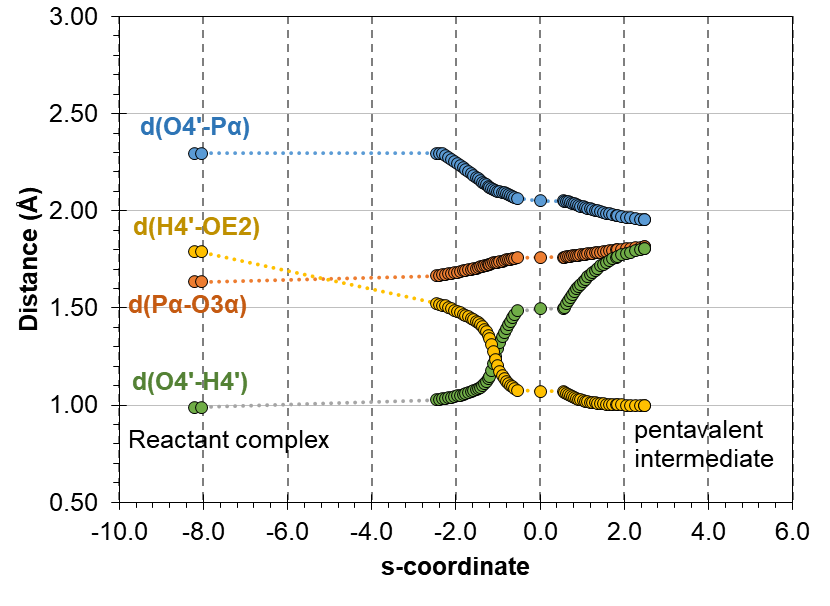
**

**Figure S5.** Free energy profile for base-assisted mechanism in aqueous solution computed using free energy perturbation method at M06-2X/6-31+G(d,p)//AMBER/TIP3P level of theory, together with evolution of key distances along reaction path.

**Table S3.** Key distances and angles for reactant complex (RC), transition state (TS) and product complex (PC) localized along ATP-assisted and Glu145-assisted mechanisms at M06-2X/6-31+G(d,p)//AMBER/TIP3P level of theory.

| Distance (Å) | **ATP-assisted** | | | **Glu145-assisted** | | |
| --- | --- | --- | --- | --- | --- | --- |
|  | RC | TS | PC | RC | TS | PC |
| Pα-O1α | 1.51 | 1.51 | 1.51 | 1.50 | 1.51 | 1.52 |
| Pα-O2α | 1.49 | 1.56 | 1.58 | 1.49 | 1.51 | 1.50 |
| Pα-O3α | 1.67 | 1.72 | 2.58 | 1.68 | 1.88 | 2.46 |
| Pα-O5’ | 1.61 | 1.61 | 1.62 | 1.60 | 1.62 | 1.61 |
| Pα-O4’ | 2.78 | 2.08 | 1.68 | 2.59 | 2.01 | 1.66 |
| O4’-H4’ | 0.98 | 1.32 | 2.09 | 1.01 | 1.33 | 2.66 |
| H4’- O2α | 2.82 | 1.12 | 1.01 | 2.86 | 2.46 | 3.14 |
| H4’- OE2^Glu145^ | 1.82 | 2.34 | 1.81 | 1.55 | 1.14 | 0.99 |
| Angle (º) |  |  |  |  |  |  |
| $∢$(O3α-Pα-O4’) | 177.9 | 166.1 | 166.3 | 174.1 | 174.7 | 168.2 |

**Table S4.** Atomic charge (in a.u.) computed for structures localized at M06-2X/6-31+G(d,p)//AMBER/TIP3P level of theory using ChelpG method.

| Atom | **ATP-assisted** | | | **Glu145-assisted** | | |
| --- | --- | --- | --- | --- | --- | --- |
|  | RC | TS | PC | RC | TS | PC |
| Pα | 1.7534 | 1.9062 | 1.9135 | 1.1350 | 1.2750 | 1.5316 |
| O1α | -1.0630 | -1.0112 | -1.0301 | -0.8525 | -0.9442 | -1.0168 |
| O2α | -1.0182 | -1.0763 | -0.9204 | -0.8953 | -0.9547 | -0.9923 |
| O3α | -0.6885 | -0.7194 | -0.8404 | -0.5838 | -0.6921 | -0.8338 |
| O5’ | -0.5178 | -0.5562 | -0.5327 | 0.0304 | 0.0045 | -0.0819 |
| O4’ | -0.8851 | -1.0187 | -0.7079 | -0.7873 | -0.8008 | -0.8187 |
| H4’ | 0.5201 | 0.6074 | 0.3664 | 0.4295 | 0.3523 | 0.1765 |
| OE2^Glu145^ | -1.0768 | -1.0511 | -1.0636 | -1.0662 | -1.0760 | -1.0653 |

**
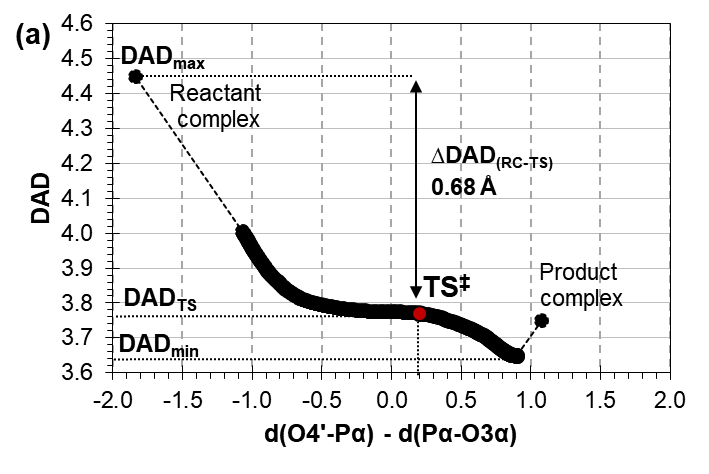

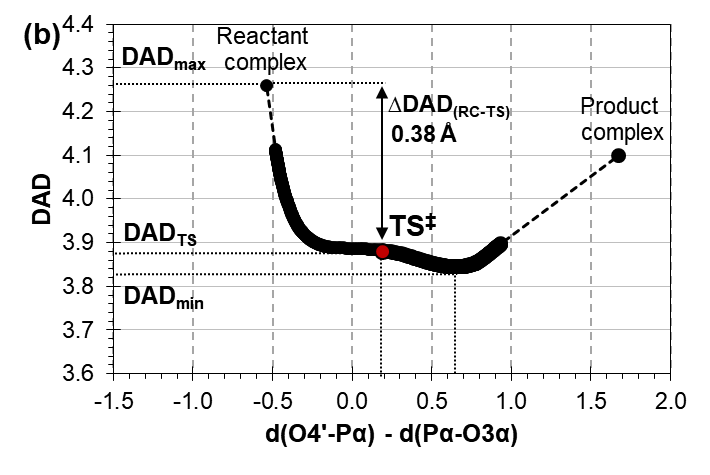
**

**
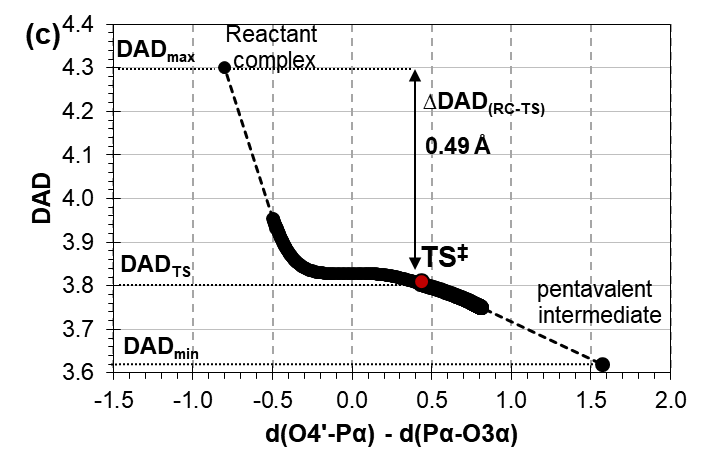
**

**Figure S6.** Evolution of donor-acceptor distance (DAD) along reaction path in (a) ATP-assisted and (b) Glu145-assisted mechanism catalyzed by ANT(4’) and (c) Base-assisted mechanism in aqueous solvent. Values of distances given in Å.

**Figure S7.** Contribution (given in %) of each amino acid residue to the overall value of the electrostatic potential generated by the enzyme on the transferred group plotted along 20ps of DFT/MM MD simulations (on the left). Structure of the active site of ANT4’ with highlighted residues with the highest favorable and unfavorable contribution to the catalytic power (on the right). Only residues that show positive or negative contribution higher than 1% are present on the plot.

**Table S5.** Geometrical coordinates of QM atoms for transition state structures localized at M06-2X/6-31+G(d,p)//AMBER/TIP3P level for reaction catalyzed by ANT(4’).

|  | **ATP-assisted** | | | | **Glu145-assisted** | | | |
| --- | --- | --- | --- | --- | --- | --- | --- | --- |
|  | Atom | x | y | z | Atom | x | y | z |
| 1 | C | -14.184 | 3.210 | -2.764 | C | -13.774 | 3.199 | -2.832 |
| 2 | H | -13.093 | 3.130 | -2.726 | H | -12.684 | 3.266 | -2.782 |
| 3 | H | -14.519 | 2.400 | -3.425 | H | -13.990 | 2.400 | -3.552 |
| 4 | C | -14.791 | 2.958 | -1.369 | C | -14.372 | 2.738 | -1.485 |
| 5 | H | -14.827 | 1.885 | -1.161 | H | -14.460 | 1.651 | -1.465 |
| 6 | H | -15.812 | 3.345 | -1.321 | H | -15.395 | 3.107 | -1.353 |
| 7 | C | -13.951 | 3.630 | -0.290 | C | -13.607 | 3.148 | -0.233 |
| 8 | O | -14.188 | 4.844 | -0.057 | O | -13.203 | 4.307 | -0.094 |
| 9 | O | -13.025 | 2.962 | 0.246 | O | -13.470 | 2.187 | 0.620 |
| 10 | C | -18.351 | 2.170 | -0.261 | C | -18.516 | 2.157 | -0.461 |
| 11 | H | -18.017 | 3.036 | -0.839 | H | -18.242 | 3.072 | -0.985 |
| 12 | H | -18.152 | 1.276 | -0.858 | H | -18.486 | 1.353 | -1.201 |
| 13 | C | -17.502 | 2.129 | 1.022 | C | -17.445 | 1.914 | 0.612 |
| 14 | O | -17.736 | 2.984 | 1.924 | O | -17.642 | 2.498 | 1.720 |
| 15 | O | -16.600 | 1.265 | 1.172 | O | -16.459 | 1.202 | 0.349 |
| 16 | C | -17.185 | -2.945 | 4.035 | C | -17.081 | -2.995 | 3.884 |
| 17 | H | -16.270 | -3.527 | 4.176 | H | -16.165 | -3.587 | 3.976 |
| 18 | H | -17.714 | -2.924 | 4.991 | H | -17.546 | -2.960 | 4.873 |
| 19 | C | -16.857 | -1.494 | 3.691 | C | -16.751 | -1.551 | 3.501 |
| 20 | H | -16.620 | -1.335 | 2.633 | H | -16.621 | -1.403 | 2.422 |
| 21 | H | -15.968 | -1.154 | 4.236 | H | -15.803 | -1.230 | 3.947 |
| 22 | C | -17.960 | -0.498 | 4.062 | C | -17.772 | -0.522 | 3.991 |
| 23 | O | -19.034 | -0.874 | 4.560 | O | -18.880 | -0.862 | 4.433 |
| 24 | O | -17.668 | 0.726 | 3.843 | O | -17.366 | 0.689 | 3.941 |
| 25 | Mg | -16.371 | 2.118 | 3.278 | Mg | -16.449 | 2.315 | 3.282 |
| 26 | O | -15.454 | 3.991 | 2.984 | O | -15.940 | 4.251 | 2.749 |
| 27 | P | -15.786 | 5.326 | 3.622 | P | -15.850 | 5.452 | 3.665 |
| 28 | O | -17.258 | 5.748 | 3.562 | O | -17.215 | 6.022 | 4.088 |
| 29 | O | -14.871 | 6.522 | 3.328 | O | -14.909 | 6.560 | 3.183 |
| 30 | O | -15.472 | 5.067 | 5.257 | O | -15.084 | 4.948 | 5.054 |
| 31 | P | -15.417 | 3.573 | 5.836 | P | -15.340 | 3.551 | 5.828 |
| 32 | O | -15.426 | 3.821 | 7.346 | O | -15.481 | 3.948 | 7.299 |
| 33 | O | -16.551 | 2.730 | 5.307 | O | -16.580 | 2.899 | 5.251 |
| 34 | O | -13.981 | 2.996 | 5.477 | O | -14.047 | 2.694 | 5.718 |
| 35 | P | -13.382 | 1.999 | 4.207 | P | -13.254 | 2.059 | 4.138 |
| 36 | O | -14.609 | 1.181 | 3.887 | O | -14.540 | 1.887 | 3.366 |
| 37 | O | -12.785 | 3.149 | 3.346 | O | -12.274 | 3.204 | 4.178 |
| 38 | O | -12.215 | 1.442 | 5.165 | O | -12.837 | 0.719 | 4.947 |
| 39 | C | -12.339 | 0.165 | 5.735 | C | -13.682 | 0.024 | 5.859 |
| 40 | H | -11.506 | -0.456 | 5.436 | H | -14.166 | -0.809 | 5.336 |
| 41 | H | -13.260 | -0.329 | 5.395 | H | -14.446 | 0.703 | 6.235 |
| 42 | C | -12.434 | 0.240 | 7.236 | C | -12.803 | -0.501 | 6.984 |
| 43 | O | -12.553 | -1.154 | 7.603 | O | -12.129 | -1.662 | 6.540 |
| 44 | C | -11.248 | -1.605 | 8.055 | C | -10.951 | -1.813 | 7.332 |
| 45 | H | -11.466 | -2.449 | 8.714 | H | -11.089 | -2.620 | 8.064 |
| 46 | C | -11.342 | 0.816 | 8.146 | C | -11.687 | 0.462 | 7.374 |
| 47 | H | -10.554 | 1.338 | 7.611 | H | -11.180 | 0.836 | 6.485 |
| 48 | O | -11.937 | 1.732 | 9.068 | O | -12.170 | 1.539 | 8.166 |
| 49 | H | -12.437 | 2.376 | 8.550 | H | -12.615 | 2.148 | 7.551 |
| 50 | C | -10.836 | -0.417 | 8.938 | C | -10.772 | -0.495 | 8.135 |
| 51 | H | -9.773 | -0.359 | 9.158 | H | -9.734 | -0.166 | 8.179 |
| 52 | O | -11.592 | -0.603 | 10.130 | O | -11.289 | -0.715 | 9.451 |
| 53 | H | -11.650 | 0.218 | 10.664 | H | -11.332 | 0.142 | 9.912 |
| 54 | C | -10.108 | -1.903 | 1.098 | C | -10.124 | -1.849 | 1.358 |
| 55 | C | -11.308 | -1.415 | 0.180 | C | -11.394 | -1.590 | 0.424 |
| 56 | C | -11.782 | -0.012 | 0.586 | C | -12.201 | -0.317 | 0.755 |
| 57 | C | -12.374 | -0.072 | 2.003 | C | -12.444 | -0.018 | 2.260 |
| 58 | C | -11.552 | -1.036 | 2.883 | C | -11.549 | -0.908 | 3.125 |
| 59 | C | -12.221 | -2.352 | 3.320 | C | -12.209 | -2.260 | 3.457 |
| 60 | N | -13.466 | -2.776 | 2.672 | N | -13.438 | -2.649 | 2.742 |
| 61 | O | -10.238 | -1.260 | 2.370 | O | -10.253 | -1.073 | 2.550 |
| 62 | O | -11.167 | -1.557 | -1.212 | O | -11.243 | -1.660 | -0.978 |
| 63 | O | -12.731 | 0.443 | -0.355 | O | -13.447 | -0.477 | 0.082 |
| 64 | O | -12.330 | 1.171 | 2.621 | O | -12.247 | 1.367 | 2.544 |
| 65 | H | -10.237 | -2.990 | 1.211 | H | -10.181 | -2.920 | 1.603 |
| 66 | H | -12.121 | -2.116 | 0.419 | H | -12.047 | -2.446 | 0.624 |
| 67 | H | -10.927 | 0.684 | 0.603 | H | -11.661 | 0.538 | 0.316 |
| 68 | H | -13.420 | -0.406 | 1.906 | H | -13.497 | -0.240 | 2.473 |
| 69 | H | -11.319 | -0.440 | 3.754 | H | -11.321 | -0.382 | 4.045 |
| 70 | H | -12.439 | -2.298 | 4.393 | H | -12.445 | -2.262 | 4.520 |
| 71 | H | -11.465 | -3.139 | 3.217 | H | -11.446 | -3.034 | 3.323 |
| 72 | H | -13.519 | -2.524 | 1.691 | H | -13.570 | -2.228 | 1.827 |
| 73 | H | -14.287 | -2.382 | 3.118 | H | -14.270 | -2.442 | 3.280 |
| 74 | H | -10.922 | -0.721 | -1.636 | H | -10.833 | -0.861 | -1.339 |
| 75 | H | -12.905 | 1.414 | -0.149 | H | -13.983 | 0.314 | 0.277 |
| 76 | H | -12.559 | 2.468 | 2.488 | H | -12.864 | 2.024 | 1.570 |
| 77 | H | -9.136 | -1.722 | 0.945 | H | -9.162 | -1.654 | 1.169 |
| 78 | H | -10.602 | -1.934 | 7.366 | H | -10.255 | -2.079 | 6.666 |
| 79 | H | -13.189 | 0.869 | 7.425 | H | -13.253 | -0.757 | 7.840 |
| 80 | H | -17.765 | -3.416 | 3.370 | H | -17.704 | -3.467 | 3.261 |
| 81 | H | -19.319 | 2.303 | -0.051 | H | -19.425 | 2.282 | -0.063 |
| 82 | H | -14.449 | 4.061 | -3.218 | H | -14.146 | 4.036 | -3.233 |

**Table S6.** Geometrical coordinates of QM atoms for transition state structure localized at M06-2X/6-31+G(d,p)//AMBER/TIP3P for reaction is aqueous solution.

|  | Atom | x | y | z |  | Atom | x | y | z |
| --- | --- | --- | --- | --- | --- | --- | --- | --- | --- |
| 1 | C | -1.356 | 0.746 | -2.346 | 34 | O | 4.228 | -1.251 | -0.683 |
| 2 | C | -0.751 | -0.558 | -3.006 | 35 | O | 3.000 | -0.610 | 1.703 |
| 3 | C | 0.264 | -1.232 | -2.105 | 36 | O | 3.423 | 1.187 | -0.212 |
| 4 | C | 1.426 | -0.326 | -1.675 | 37 | C | 4.456 | 2.029 | -0.677 |
| 5 | C | 0.957 | 1.149 | -1.731 | 38 | H | 4.702 | 1.794 | -1.720 |
| 6 | C | 1.207 | 1.919 | -3.050 | 39 | H | 5.375 | 1.901 | -0.099 |
| 7 | N | 2.592 | 2.026 | -3.501 | 40 | C | 3.988 | 3.453 | -0.497 |
| 8 | O | -0.429 | 1.246 | -1.390 | 41 | O | 2.786 | 3.716 | -1.215 |
| 9 | O | -1.636 | -1.585 | -3.472 | 42 | C | 2.201 | 4.895 | -0.656 |
| 10 | O | 0.671 | -2.372 | -2.858 | 43 | H | 2.525 | 5.779 | -1.224 |
| 11 | O | 1.825 | -0.734 | -0.388 | 44 | C | 3.556 | 3.750 | 0.941 |
| 12 | H | -1.488 | 1.473 | -3.164 | 45 | H | 2.872 | 2.954 | 1.245 |
| 13 | H | -0.214 | -0.219 | -3.900 | 46 | O | 4.613 | 3.914 | 1.864 |
| 14 | H | -0.247 | -1.557 | -1.182 | 47 | H | 5.024 | 3.053 | 2.040 |
| 15 | H | 2.251 | -0.473 | -2.397 | 48 | C | 2.776 | 5.054 | 0.772 |
| 16 | H | 1.464 | 1.681 | -0.930 | 49 | H | 2.006 | 5.185 | 1.537 |
| 17 | H | 0.816 | 2.928 | -2.884 | 50 | O | 3.663 | 6.160 | 0.773 |
| 18 | H | 0.633 | 1.506 | -3.883 | 51 | H | 4.354 | 5.962 | 1.421 |
| 19 | H | 2.906 | 1.144 | -3.895 | 52 | Mg | 5.854 | -2.229 | -0.828 |
| 20 | H | 3.165 | 2.212 | -2.689 | 53 | C | 1.861 | -6.853 | -0.950 |
| 21 | H | -1.254 | -2.420 | -3.160 | 54 | H | 1.090 | -7.015 | -0.193 |
| 22 | H | 1.347 | -2.834 | -2.317 | 55 | H | 1.687 | -7.549 | -1.776 |
| 23 | H | 2.058 | -2.210 | -0.435 | 56 | C | 1.835 | -5.417 | -1.457 |
| 24 | O | 5.946 | -3.572 | 0.523 | 57 | H | 0.891 | -5.216 | -1.983 |
| 25 | P | 6.529 | -3.335 | 1.933 | 58 | H | 2.623 | -5.247 | -2.197 |
| 26 | O | 7.978 | -3.808 | 2.031 | 59 | C | 1.957 | -4.353 | -0.369 |
| 27 | O | 5.663 | -3.814 | 3.077 | 60 | O | 1.836 | -4.585 | 0.815 |
| 28 | O | 6.544 | -1.677 | 2.188 | 61 | O | 2.173 | -3.163 | -0.911 |
| 29 | P | 6.677 | -0.372 | 1.228 | 62 | H | 2.827 | -7.084 | -0.495 |
| 30 | O | 7.523 | 0.653 | 1.922 | 63 | H | 1.212 | 4.783 | -0.756 |
| 31 | O | 7.104 | -0.884 | -0.160 | 64 | H | 4.697 | 4.082 | -0.817 |
| 32 | O | 5.189 | 0.198 | 1.128 | 65 | H | -2.194 | 0.794 | -1.804 |
| 33 | P | 3.672 | -0.308 | 0.392 |  |  |  |  |  |
